# Mitophagy in Human Diseases

**DOI:** 10.3390/ijms22083903

**Published:** 2021-04-09

**Authors:** Laura Doblado, Claudia Lueck, Claudia Rey, Alejandro K. Samhan-Arias, Ignacio Prieto, Alessandra Stacchiotti, Maria Monsalve

**Affiliations:** 1 Instituto de Investigaciones Biomédicas “Alberto Sols” (CSIC-UAM), Arturo Duperier 4, 28029 Madrid, Spain; lauradoblado@iib.uam.es (L.D.); claudialueck@hotmail.de (C.L.); claudiareycc@gmail.com (C.R.); 2 Department of Biochemistry, Universidad Autónoma de Madrid e Instituto de Investigaciones Biomédicas “Alberto Sols” (CSIC-UAM), Arturo Duperier 4, 28029 Madrid, Spain; alejandro.samhan@uam.es; 3 Instituto de Investigación Sanitaria de la Fundación Jiménez Díaz, Isaac Peral 42, 28015 Madrid, Spain; nprieto58@gmail.com; 4 Department of Biomedical Sciences for Health, Universita’ Degli Studi di Milano, Via Mangiagalli 31, 20133 Milan, Italy; 5 U.O. Laboratorio di Morfologia Umana Applicata, IRCCS Policlinico San Donato, San Donato Milanese, 20097 Milan, Italy

**Keywords:** mitophagy, Parkin, PINK1, aging, Parkinson’s, Alzheimer’s, Huntington’s, dementia, diabetes, atherosclerosis, heart failure, muscle wasting, exercise, mice, rats

## Abstract

Mitophagy is a selective autophagic process, essential for cellular homeostasis, that eliminates dysfunctional mitochondria. Activated by inner membrane depolarization, it plays an important role during development and is fundamental in highly differentiated post-mitotic cells that are highly dependent on aerobic metabolism, such as neurons, muscle cells, and hepatocytes. Both defective and excessive mitophagy have been proposed to contribute to age-related neurodegenerative diseases, such as Parkinson’s and Alzheimer’s diseases, metabolic diseases, vascular complications of diabetes, myocardial injury, muscle dystrophy, and liver disease, among others. Pharmacological or dietary interventions that restore mitophagy homeostasis and facilitate the elimination of irreversibly damaged mitochondria, thus, could serve as potential therapies in several chronic diseases. However, despite extraordinary advances in this field, mainly derived from in vitro and preclinical animal models, human applications based on the regulation of mitochondrial quality in patients have not yet been approved. In this review, we summarize the key selective mitochondrial autophagy pathways and their role in prevalent chronic human diseases and highlight the potential use of specific interventions.

## 1. History and Pathways of Mitophagy

The capacity of the eukaryotic cell to regulate mitochondrial function provides the organisms with key metabolic plasticity, essential for a wide variety of cell functions [1,2]. Hence, maintenance of mitochondrial function relies on the adequate co-regulation of functions that control their turnover, namely mitochondrial biogenesis, which produces new mitochondria and mitophagy which eliminates damaged or unnecessary mitochondria [3]. Insufficient mitophagy leads to the accumulation of poorly functional/damaged mitochondria, with a reduced capacity to synthesize Adenosine triphosphate (ATP^+^), that produce high levels of superoxide. This can result in alteration in the cellular pools of intermediate metabolites, with pathological consequences [4]. Poorly functional mitochondria are a well-known hallmark of metabolic and neurodegenerative diseases, which are strongly linked to pathological developments. Alterations in the activity of key mitophagy regulators are central to these processes.

### 1.1. Mitophagy, a Type of Autophagy

It has to be highlighted that mitophagy is a type of selected autophagy [5]. Autophagy, literally “the process of the cell eating itself” in Greek, is divided into micro- or macro-autophagy, and chaperone-mediated autophagy, depending on the size of the degraded structure, and can be nonselective or selective, depending on whether any specific cellular component is targeted [6]. Of note, non-selective autophagy is emerging as a primary mechanism in cell death [7]. Early studies suggested that selective autophagy was closely related to (non-selective) macroautophagy, the only apparent difference being an additional step targeting isolation membranes to cargo. However, it has now been well established that, at least in yeast, several components of the canonical macroautophagy pathways are often dispensable for selective autophagy [8]. Therefore, mitophagy is a selective autophagy process that involves isolation within a membrane, sealing, and degradation through the lysosomal pathway of the organelle [9]. However, most subcellular structures, not just mitochondria, are targets of selective autophagy, including Golgi, the endoplasmic reticulum (ER), peroxisomes, ribosomes, the midbody, lipid droplets, and glycogen granules.

Mitophagy, defined as the selective autophagy of damaged mitochondria, was firstly described in yeast, where the presence of a mutated Uth1p in the outer mitochondrial membrane (OMM) was found to block autophagy during starvation [10]. Similar findings were later reported in cultured starved hepatocytes that eliminated damaged mitochondria when exposed to oxidative damage [11]. Commonly, the morphological characteristic feature of mitophagy is considered to be the localization of mitochondria inside an autophagic vacuole, called mitophagosomes [11,12]. However, currently, it is considered that there are three types of mitophagy: type 1, induced by nutrient limitation, type 2, induced by damage signals, and type 3, micro-mitophagy, linked to small mitochondria-derived vesicles [13]. These processes are intrinsically different, because type 1 and type 2 require the fusion of a lysosome to produce an autophagosome encircling mitochondria, while the latter type does not. Mitophagy plays a relevant role in normal development, as recently analyzed and quantified in vitro and in vivo in fluorescent transgenic mouse models (like mt-Keima or mito-QC) [14,15]. However, more generally, this fundamental biological mechanism works in all cells or tissues, being regulated in response to their changing energetic requirements. Some tissues, such as the nervous system, the kidney, the skeletal muscle, the heart, and the liver, show high basal mitophagy activity, while others, such as the spleen and the thymus, display low mitophagy levels [15,16]. The molecular and biochemical pathways involved in mitophagy were first characterized in models of aging [4], neurodegenerative and psychiatric diseases [17], cancer [18], and cardiovascular diseases (CVD) [19]. Basal mitophagy is, for example, vital to maintain synaptic plasticity and to eliminate damaged mitochondria in the brain, while its deranged activity is associated with age-related neuronal damage [20,21].

### 1.2. PINK1

Mitophagy can be phosphatidylinositol-3,4,5-trisphosphate 3-phosphatase (PTEN)-induced putative kinase 1 (PINK1)-dependent or -independent [22]. PINK1 is a serine/threonine kinase whose levels are normally low, but it is stabilized and accumulates at the OMM in response to mitochondrial damage (mtDNA mutations), increased mitochondrial reactive oxygen species (ROS), depolarization, and the accumulation of misfolded proteins [23]. Accumulated PINK1 is autophosphorylated and activated, and in turn phosphorylates ubiquitin on serine 65, which recruits Parkin from the cytosol to the mitochondrial membrane. Parkin is an E3-ubiquitin ligase that, when recruited and activated, drives the ubiquitination of mitochondrial proteins and hence autophagy [24,25,26]. Recently, an inhibitory mechanism of the pathway has been described, and Ubiquitin carboxyl-terminal hydrolase 30 (USP30) can act as a brake on mitophagy by opposing Parkin-mediated ubiquitination [27]. Importantly, although PINK1 facilitates Parkin recruitment, Parkin can be recruited to depolarized mitochondria and drive mitophagy even in the absence of PINK1 [28]. Some identified targets of Parkin ligase activity at the OMM include Mitofusin 1 and 2 (MFN1/2), voltage dependent anion channel protein 1 (VDAC1), and mitochondrial Rho guanosine triphosphate hydrolases (GTPases) (MIRO) [28]. However, a widespread degradation of OMM has been evidenced by proteomic studies, suggesting that remodeling of the mitochondrial outer membrane proteome is important for mitophagy [26].

Under physiological steady state conditions, PINK1 is imported into the mitochondria through the translocase of the outer mitochondrial membrane (TOMM) complex of the OMM and into the translocase complex (TIMM) of the inner mitochondrial membrane (IMM), where it is cleaved by the mitochondrial processing peptidase (MPP) [29]. Afterwards, PINK1 is also cleaved in its hydrophobic domain, spanning the IMM, by the rhomboid protease presenilin-associated rhomboid-like protein (PARL), generating a 52 kD, N-terminal-deleted form of PINK1 [30]. PARL cleavage releases this PINK1 into the cytosol, where it is targeted by the N-degron type-2 E3 ubiquitin ligases and degraded by the ubiquitin proteasome system (UPS) [31]. This import and degradation cycle maintains PINK1 at very low, almost undetectable, levels on healthy mitochondria. However, mitochondrial import, through the TIMM complex, is affected by membrane depolarization, inhibition of the electron transport chain, genetic or environmental stressors, such as inflammation, and the accumulation of unfolded proteins. Under these adverse conditions, PINK1 processing by PARL is prevented, and uncleaved PINK1 accumulates on the OMM, bound to the TOMM complex [29]. This last event is needed to target PINK1 to selected single damaged mitochondrion [32].

### 1.3. Mitochondrial Homeostasis-Related Pathways

It should be highlighted that mitochondrial control through mitophagy is actually part of a more complex homeostatic control process of mitochondria that includes fusion and fission dynamics and mitochondrial biogenesis, with all these processes being interregulated [33]. Of note, mitochondrial fusion is induced upon starvation, and fused mitochondria are particularly resistant to mitophagy, while fragmented/fused organelles with low membrane potential (Δψ_m_) are more easily targeted into mitophagosomes [34,35]. Accordingly, mitochondrial fusion/fission regulatory cues are also mitophagy modulators [36]. Other regulatory pathways still need to be fully characterized; for example, it has been suggested that mitophagy selectively targets certain mitochondria based on their topology. A recent study reported that serum-starved U2OS osteosarcoma cells formed “donut” mitochondria that exhibited normal inner membrane potential (Δψ_m_) and were resistant to mitophagy, while swollen mitochondria with low potential were removed [37]. Mitophagy has also been shown to be regulated by changes in mitochondrial subcellular location and changes in cellular bioenergetics through regulators that control the main anabolic and catabolic pathways, as well as mitochondrial biogenesis [38]. 

Guanosine triphosphate hydrolases (GTPases) Mitofusin 1 and Mitofusin 2 (MFN1/2) are key players in the control of mitochondrial dynamics (fusion and fission) and orchestrate mitochondrial network connectivity and activity [39]. When mitochondria oxidative phosphorylation (OXPHOS) is activated, they fuse into a network that can cover the whole cell. Conversely, inhibition of mitochondrial OXPHOS activity is linked to the breakdown (fission) of the network into small mitochondrial units that tend to localize close to the nuclei. Fusion is induced by homo or hetero dimerization of MFN1/2, anchored to OMM at their C-termini, which mediate the GTP-dependent merge of separate OMMs. Fusion is also activated by MitoPLD, a member of the phospholipase D family, which converts, the mitochondrial-specific lipid cardiolipin (CL) into phosphatidic acid. CL is predominantly localized into the IMM, but mitochondrial damage leads to its relocalization to the OMM [40]. Fusion of the IMM and cristae organization requires full-length Optic Atrophy Protein 1 (L-OPA1). In cellular stress conditions, L-OPA1 is cleaved to S-OPA1, promoting OMM permeabilization and cytochrome *c* release [41]. The fission of mitochondrial OMM is also regulated by another GTPase protein, called dynamin-related protein 1 (Drp1) and its receptor proteins fission protein 1 (Fis1), mitochondrial fission factor (Mff) and mitochondrial dynamic proteins 49 and 51 kDa (MiD49 and MiD51) [42]. Intracellular signaling pathways regulate the positioning of Drp1 on the OMM. Once recruited, Drp1 oligomerizes into a ring-like structure that wraps around the mitochondria, which is also marked by the presence of endoplasmic reticulum (ER) and actin cytoskeleton, constricts the mitochondrial membrane and triggers fission [43].

Several related pathways have now been found to link mitochondrial dynamics to mitophagy, since damaged or unnecessary mitochondria should first be fused out and then degraded. In particular, MFN1/2 are extracted from the OMM by a ubiquitin-dependent chaperone and degraded by the proteasome [44]. Ubiquitination and depletion of MFN1/2 prevents the fusion of damaged mitochondria and leads to fragmentation, as fission processes remain functional, which promotes mitophagy [45]. PINK1 phosphorylates MFN2 that then works as a Parkin receptor for culling damaged mitochondria [46].

Although not a necessary element, voltage-dependent anion-selective channel 1 (VDAC1) also plays a relevant role in the control of mitophagy. VDAC1, the most abundant OMM protein, can be considered a mitochondrial porin. It largely controls mitochondrial permeability to a number of metabolites across the OMM and is a key regulatory element in mitochondria-dependent apoptosis [47]. It has been shown to interact with Parkin and become ubiquitinated and to be involved in Parkin recruitment. A recent study on VDAC1′s role in mitophagy revealed that Parkin can induce both mono- and polyubiquitination on VDAC1 [48]. Conversely, defective monoubiquitination leads to the induction of apoptosis, and reduced polyubiquitination hinders mitophagy, suggesting that VDAC1 interaction with Parkin is at a crossroads in terms of the decision to induce mitophagy or apoptosis by damaged mitochondria [49]. Of interest, another study identified an additional functional pathway of VDAC1 in mitophagy control through the cholesterol translocator protein (TSPO) [50]. TSPO facilitates the transfer of cholesterol from the OMM to the IMM, where it serves as a precursor for the synthesis of steroid hormones. It forms a functional complex with VDAC1 and has been shown that its overexpression inhibits mitophagy though an ROS-dependent mechanism that did not prevent the recruitment of Parkin but blocked the ubiquitination of mitochondrial proteins, though a still undefined mechanism.

Parkin also ubiquitinates the mitochondrial outer membrane Rho GTPases (MIRO1/2), which directly interact with PINK1 [51]. These proteins are components of the adaptor complex that anchors mitochondria to motor proteins. Thus, they are involved in the regulation of axonal mitochondrial movement by Ca^2+^ [52]. When Ca^2+^ binds, it causes the dissociation of motor/adaptor complexes from microtubules, thus leading to a mitochondrial movement arrest that facilitates the removal of damaged mitochondria by mitophagy [53]. MIRO serves as a Ca^2+^-dependent docking site and directly primes Parkin recruitment. However, the role of PINK1 and Parkin in MIRO1 degradation remains controversial. In fact, it has been proposed that MIRO1 ubiquitination, rather than its degradation, is the main signal for mitochondrial arrest [54].

### 1.4. LC3

Ubiquitination of the cargo is a critical step in selective autophagy in all cases [55]. The most accepted model is that cargo-bound receptors recruit microtubule-associated protein 1 light chain 3 (LC3) through an LC3-interacting region (LIR), bridging cargo with a preformed, autophagy-generated membrane. In this model, receptors are either integral to the cargo or recruited to the cargo via ubiquitination. A scaffold protein, which recruits additional autophagy-related proteins, may also be involved [56].

In mitochondria, following OMM remodeling mediated by proteasomal degradation of ubiquitinated proteins, adaptor proteins that bind ubiquitin (Ub) are recruited for the transport of depolarized mitochondria to the perinuclear region through a microtubule-dependent mechanism [57]. These adaptors interact with microtubule-associated protein 1 light chain 3 (LC3), which in turn promotes the sequestration of damaged mitochondria into autophagosomes. Finally, the autophagosomes fuse with the lysosomes, leading to the degradation of damaged mitochondria [5]. Five mitochondrial cargo-bound receptors (LC3 adapters) that contain an LIR motif that is recognized by LC3 [58] are recruited to the polyubiquitinated substrates on the mitochondria through their ubiquitin-binding domain: sequestosome-1 (p62), optineurin (OPTN), nuclear domain 10 protein 52 (NDP52), Trans-activating transcriptional regulatory protein of HTLV-1 (TAX1) binding protein 1 (TAX1BP1), and neighbor of Breast Cancer 1 (BRCA1) gene 1 (NBR1). Of note, it has been shown that OPTN [59] is largely dependent on its activation by Tank-binding kinase 1 (TBK1), a key signaling regulator of innate immunity, which highlights the interplay between mitophagy and the regulation of the immune system [60].

### 1.5. Ubiquitin Independent Mitophagy

It has been demonstrated that autophagy and mitophagy are upregulated in cells lacking PINK1 [61]. Damaged mitochondria can also be recognized by LC3 adapters in a ubiquitin-independent manner. These adapters directly sense mitochondrial damage and consequently change their subcellular location or the protein they interact with, guiding the damaged mitochondria to the autophagosome. The best characterized systems involved in the programmed mitochondrial clearance or mitochondrial elimination in the context of a developmental program are the B-Cell CLL/Lymphoma 2 (BCL2)/adenovirus E1B 19 kDa-interacting protein 3 (BNIP3) and BCL2/adenovirus E1B 19 kDa-interacting protein 3-like (NIX/BNIP3L) pathways [62]. The current evidence suggests that both BNIP3 and NIX play an important role in oxygen sensing, inducing mitophagy in response to hypoxia, and can also directly promote the depolarization of mitochondria, as well as the fusion with cellular membranes. BIP3 and its homolog NIX are transmembrane OMM proteins. Their cytoplasmic N-terminal portion can interact with LC3-related molecules, targeting mitochondria for degradation by autophagy. BNIP3 is able to interact directly with PINK1, stabilizing it and promoting its ability to recruit Parkin, and its activity involves Drp1-mediated mitochondrial fission [63].

Other Parkin-independent mechanisms include those mediated by receptors, such as FUN14 domain-containing protein 1 (FUNDC1), another mitochondrial OMM protein sensitive to hypoxia [64]. Choline dehydrogenase (CHDH) is located in the IMM and OMM under normal conditions. When the mitochondrial membrane potential is disrupted, CHDH accumulates in the OMM and interacts with p62 through its Phox and Bem1 (PB1) domain, leading to the formation of the CHDH-p62-LC3 complex that mediates mitophagy [65]. TBC1 domain family member 15 (TBC1D15), a mitochondrial Rab GTPase activating protein, forms a complex with TBC1D17 and migrates to the mitochondrial outer membrane by interacting with Fis1. The TBC1D15/17 complex then interacts with LC3 [66]. Bcl2 like 13 (BCL2L13) is the mammalian homologue of Autophagy-related protein 32 Atg32, the only mitophagy receptor found in yeast [67]. Like other LC3 receptors, BCL2L13 locates on the OMM and binds to LC3 via the LC3-interacting region. FK506-binding protein 8 (FKBP8), located on the OMM, was identified as an LC3 interacting protein using yeast two-hybrid screening [68]. Remarkably, specific IMM components have also being shown to participate in mitophagy. Prohibitin 2 (PHB2) is a IMM protein [69] that becomes exposed to LC3 following Parkin-mediated degradation of OMM proteins. CL, as mentioned above, a membrane lipid in the IMM, can also function as an LC3 receptor in mitophagy when translocated from the IMM to the OMM in the presence of external depolarizing toxins [70]. Of note, the nutrient deprivation sensor, adenosine monophosphate activated protein kinase (AMPK), has also been shown to induce Parkin-independent mitophagy through the phosphorylation and activation of TBK1 [71].

**Figure 1:**
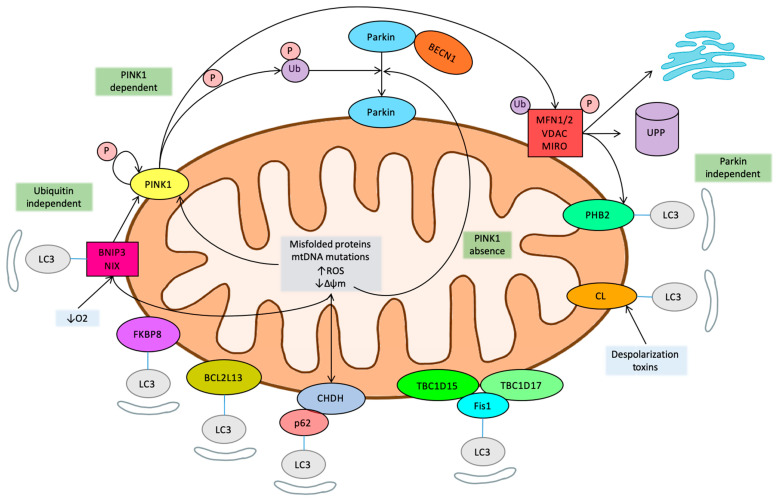
Summary of main mitophagy pathways.

The mitophagy main regulatory pathways have been summarized in Figure 1.

### 1.6. Novel Regulatory Pathways

Recent studies have also shown the physiological relevance of LC3-independent mitophagy. In particular, mitophagy can be driven by Rab9-associated autophagosomes, through the formation of a protein complex that involves Rab9, Unc-51-like kinase 1 (ULK1), and Drp1 [72].

Additional, novel pathways that impact mitophagy continue to be identified almost daily. For example, it has been demonstrated that, several ligases may regulate mitophagy, such as SMAD-specific E3 (SMURF1) [73], while the autophagy protein Coiled-coil myosin-like BCL2-interacting protein (BECN1)/Beclin 1, which plays a central role in autophagosome formation and maturation, has been shown to interact with Parkin and does not require its translocation to mitochondria [74].

Recently, a number of studies have focused on evidence linking mitophagy to ER stress, through the specialized ER-mitochondrial contact regions (MAMs) that regulate Ca^2+^ fluxes and control the induction of apoptosis [75]. PINK1 controls mitochondrial Ca^2+^ efflux [76,77], while, in turn, PINK1 gene expression has also been shown to be sensitive to Ca^2+^ fluxes [78]. The role of MAMS as key regulators of mitophagy is now well established, as they have been shown to be indispensable in the autophagy process, with many proteins that are directly involved in autophagy located in MAMs. In fact, in response mitophagy stimuli PINK1 and Beclin 1 have been shown to relocalize at MAMs where they further promote the association of mitochondria with ER, and autophagosome formation [79]. Although the mechanisms involved remain to be clearly elucidated [80], its physiological relevance has been clearly demonstrated, particularly in the context of Parkinson’s disease [51,81].

## 2. Mitophagy in Neuropsychiatric and Neurodegenerative Diseases

Aberrant mitophagy is implicated in the pathogenesis of several neurodegenerative, cardiovascular, metabolic, and skeletal muscle diseases [82], while the beneficial effects of targeting molecules, such as urolithin A and actinomycin, have been reported in old mice, mouse models of Alzheimer’s disease, and other preclinical rodent models of neurodegenerative and cardiovascular diseases [83].

Alterations in mitochondrial number and activity have been identified in a wide variety of neuropsychiatric and neurodegenerative diseases. However, the adequate evaluation of the causes and impact on disease development is still obscure in most cases. In general, the accumulation of damaged mitochondria suggests that the process of mitophagy might be dysregulated. The evidence so far supports this general concept, although how the observed changes affect disease development is not fully elucidated and seems to be drastically dependent on the specific pathological context. In general terms though, it has been proposed that the upregulation of mitophagy in neurodegenerative diseases, contrary to cardiovascular diseases, can be beneficial, and might even be essential, for the well-being of neurons [84,85]. 

### 2.1. Mitophagy in Neurodegenerative Diseases (Parkinson’s, Dementia, Alzheimer’s, Post-Stroke Cognitive Impairment)

#### 2.1.1. Parkinson’s Disease

Parkinson’s disease (PD) is represented by bradykinesia, tremors, rigidity, postural instability, and other symptoms, such as an altered perception of smell, constipation, and depression. Pathological markers of this disease are seen in the *substantia nigra* of the *pars compacta*, manifesting in a loss of dopaminergic neurons and the presence of Lewy bodies, formed by aggregates of α-synuclein, in both genetic and sporadic Parkinsonism [86]. 

PD, characterized by a specific loss of dopaminergic neurons, was the first neurodegenerative disease about which the presence and significance of mitochondrial malfunction was described. PD patients consistently showed reduced and altered activity of the Complex I of the electron transport chain (ETC) [87], along with an increased production of mitochondrial ROS [88]. Early on, a number of studies also suggested an altered mitophagy after finding mitochondria in human neuronal autophagosomes and, later on, abnormal mitophagy in sporadic and hereditary PD. Furthermore, when the genetic basis of early onset PD was established, key factors controlling mitochondrial hormesis were discovered, in particular mutations in *PARK6* and *PARKIN*, genes encoding PINK1 and Parkin [32,89]. In fact, PINK1/Parkin-mediated mitophagy are the main focus of a large number of studies on PD [90]. Parkin levels are under strict transcriptional control by several transcription factors, such as sterol regulatory element binding transcription factor 1 (SREBF1) and F-box and WD40 domain protein only protein 7 (FBXW7). Importantly, SREBF1 has also been shown to be a risk locus for sporadic PD [91,92].

Recent studies aimed at evaluating the relevance of the observed impairment of mitophagy in PD have further evidenced that the process is altered at multiple levels [93]. Ubiquitination of mitochondria by Parkin is insufficient, resulting in failed recognition by p62 and OPTN, since changes in PINK1 and Parkin impact MFN1/2, regulators of mitochondrial dynamics [94]. Remarkably, a loss of either PINK1 or Parkin activity results in an accumulation of both MFNs, impairing mitophagy through a low recruitment of ubiquitin-binding proteins and over-enhanced mitochondrial fusion [95]. Parkin may influence the mitochondrial ETC by interacting with Stomatin-like protein 2 (SLP-2), a protein necessary for the assembly of the ETC [96], and regulates mtDNA transcription by the upregulation of Peroxisome proliferator-activated receptor gamma coactivator 1-alpha (PGC-1α). Additionally, mutant PINK1 can directly inhibit ETC Complex I [97]. Therefore, when Parkin is mutated in PD, OXPHOS activity is highly compromised. These changes can also be related to the observed alterations on the activity of sirtuins in the context of PD [98].

Recently, mutations of F-box only protein 7 (FBXO7), an adaptor protein in Skp-Cullin-F-box ubiquitin E3 ligase complex (SCF complex), were found to induce early-onset juvenile autosomal recessive PD with rapidly progressive and serious PD symptoms (Parkinson disease-15, PARK15) [99]. The F-box proteins serve as adaptor proteins in the SCF complex to recognize its substrates (usually in phosphorylated status) via interaction with SKP1 protein, so as to facilitate the ubiquitination of substrates by adjacent ubiquitin E2 conjugating enzyme recruited by the Ring-Box 1 (RBX1) protein [100]. It has been shown that FBXO7 is a stress-responsive protein that translocates from the nucleus to mitochondria, where it can form protein aggregates, whose formation is enhanced by mutations linked to the development of PD and by damaged mitochondrial ROS production, thus impairing mitophagy [101]. Importantly, recent studies support a direct, protective role of Wild Type (WT) FBXO7 activity from PD development. It was demonstrated that WT FBXO7 directly interacts with Parkin and can rescue PD development in a Parkin mutant model by promoting mitophagy, while pathogenic FBXO7 mutants inhibit mitophagy [102,103].

Mutations in *PARK7*, which encodes the protein Parkinsonism Associated Deglycase (DJ-1), cause early-onset recessive PD [104]. DJ-1 was initially described as a regulator of cell death and later shown to also be involved in dopamine oxidation, mitochondrial and lysosome dysfunction in the context of PD. DJ-1 is found as a homodimer in the cytosol in basal conditions, but, in response to stress stimuli, it re-localizes to other cellular compartments, including mitochondria [105], where it induces mitochondrial fission. Some DJ-1 mutants found in PD can still translocate to mitochondria but do not show its protective regulatory activities, leading to impaired mitochondrial dynamics and function [106]. It has been shown that DJ-1, when upregulated, also affects macroautophagy, while the loss of DJ-1 blocks basal autophagy and impairs mitochondrial dynamics [107]. In line with those results, it has been suggested that DJ-1 may induce the selective removal of damaged mitochondria in response to stress, possibly through the direct interaction of DJ-1 with PINK1. DJ-1 has also been proposed to regulate chaperone-mediated autophagy by preventing protein aggregation, thereby aiding amelioration of PD progression. This activity could be related to the capacity of DJ-1 to repair glycated forms of protein and DNA [108]. Likely on the same function, DJ-1 has been shown to be a regulator of ubiquitin-independent 20S proteasomal degradation, supporting its key role in the maintenance of protein homeostasis, a key hallmark of several neuronal disorders, including PD [109].

Autosomal dominant mutations in *LRRK2/PARK8* are the cause of both familial and sporadic PD [110,111]. The precise function of LRRK2, Leucin-rich repeat serine/threonine kinase2, remains unknown, but various roles in vesicle synthesis and trafficking, including roles related to autophagy and to the regulation of mitochondrial homeostasis, have been proposed [112,113]. LRRK2 is related to various mitochondrial processes, including fission, apoptosis through changes in Bcl-2 phosphorylation [114], and intracellular transport, through the control of MIRO1 degradation [115]. The loss of LRRK2 causes dysfunctional autophagy and an accumulation of autophagosomes, whereas increments in LRRK2 cause deficiencies in chaperone-mediated autophagy (CMA) by increasing the lysosomal binding of LRRK2, resulting in interference with the CMA translocation complex [113,116]. To control vesicular transport at the synaptic terminal, LRRK2′s WD40 domain binds and sequesters synaptic vesicles. In PD, the binding affinity is reduced by mutations in the WD40 domain, and vesicular trafficking is impaired as a result [117] LRRK2 also regulates autophagy through the activation of a Ca^2+^-dependent protein kinase kinase-β (CaMKK-β)/adenosine monophosphate activated protein kinase (AMPK) pathway, resulting in an increase in autophagosome formation [118]. Some LRRK2 mutations in PD show increased kinase activity [119], which activates the autophagy receptor p62 but decreases the number of lysosomes with an acidic pH, resulting in the accumulation of autophagosomes and impaired autophagy [120].

The death of dopaminergic neurons is preceded by the formation of intracellular aggregates known as Lewy bodies, which contain a variety of misfolded protein components, but are mostly made up of α-synuclein (α-syn) and ubiquitin [121]. Importantly, pathological mutations in α-syn are found in both familiar and idiopathic forms of the disease [122]. Although aggregated α-syn was first considered the main pathogenic driver of the disease, accumulated evidence suggests that macroaggregates are an attempt to sequester aberrant proteins, whereas soluble oligomers of pleated β-sheets (micro-aggregates) are the most toxic forms. In fact, pathological mutations of α-syn accelerate β-sheet formation and fibrilization [123].

Since α-syn tangles are the hallmark of PD, a significant number of studies have identified links between α-syn and mitochondrial function. It is now well established that compromised mitochondrial activity, reduced ATP output, and increased superoxide production impairs proteostasis, leading to the accumulation of oxidatively modified α-syn [124].

In line with these findings are studies on mutations in ATP13A2, which cause a complicated form of autosomal recessive PD [125]. ATPase13A2 is a lysosomal type 5 P-type ATPase that has been proposed to function as a cation pump and localizes multi-vesicular bodies. When mutated, it impairs mitochondrial function and, as a result, hampers the exosomal release of α-syn [126]. Of note, ATPase13A2 has been functionally related to another PD associated gene coding for Synaptotagmin 11 [127].

Another gene mutated in genetic PD that links vesicular traffic, mitochondrial function, and α-syn is VPS35, a component of the retromer, a protein complex that is associated with the endosome to facilitate both the endosome-to-Golgi complex and the endosome-to-plasma membrane transport or recycling of transmembrane protein cargo, which is responsible for vesicular transport from the Golgi apparatus to the endosome. Its PD-linked mutations both influence ETC Complex I activity and α-syn accumulation [128].

Furthermore, α-syn is normally degraded in concert with the activation of mitochondrial fission and Parkin [129]. Therefore, when Parkin activity is reduced, α-syn accumulates and impairs mitochondrial function that links α-syn to mitophagy [130].

More recently, 𝛼-syn has also been demonstrated to directly regulate mitochondria [131], interacting with membrane acidic phospholipids at MAMs and altering their morphology and function [132]. Nuclear magnetic resonance studies have suggested that α-syn is intrinsically disordered and that its interaction with negatively charged phospholipids in membranes promotes the adoption of an α-helical structure. The association of α-syn with membranes is altered by α-syn mutations found in several rare familial forms of PD. Upon binding to mitochondria, α-syn can contribute to mitochondrial depolarization and fragmentation. Overexpressed mutant α-syn binds to TOMM20, which in turn inhibits the interaction with TOMM22, which is a necessary step during mitochondrial protein import, and leads to ETC malfunction. This augments ROS levels and DNA damage and facilitates apoptosis induced by cyt *c* release.

In the mitochondria, mutant α-syn also binds CL, increasing LC3 recruitment and thus increasing mitophagy [133]. However, this is insufficient to compensate the accumulation of damaged mitochondria and the deficits in other mitophagy-related pathways found in PD. Of note, CL has been proposed to play a proteostatic protective role in PD, since OMM-localized CL can pull α-syn monomers away from oligomeric fibrils and facilitate their refolding from aggregated β-sheet forms back to monomers comprising α-helices [123]. 

It should also be highlighted that, whereas recent genetic discoveries have led to a number of different genetic models of PD, they failed to reproduce the broad extranigral pathology and other pathological landmarks of PD, while models using environmental pesticides, long considered as risk factors for PD, better recapitulate the human disease. Other than age and genetic background, the possible role of the exposure to some pesticide and metals as drivers of the disease has been the focus of research for a number of years, but proof of a causative relationship has remained elusive. The strongest evidence is still the observation that PD patients consistently show reduced ETC Complex I activity and that both the chemical and genetic models of PD are all associated with mitochondrial dysfunction [134]. In addition, while even the most recent advanced genetic models fail to find the basis for PD specific sensitivity [135], the old concept that focused on the alterations in dopamine mitochondrial metabolism and toxicity stays unchallenged but almost forgotten [136].

#### 2.1.2. Dementia

Dementia is often used as a generic term for symptoms concerning severe decline in cognitive function and social abilities, such as memory, judgement, and sometimes language. It can be subtyped into different forms, the most common being Alzheimer’s disease (AD) [137]. Other common forms of dementia include Frontotemporal Dementia (FTD), vascular dementia, and human immunodeficiency virus (HIV)-associated neurocognitive disorder and Lewy body dementia [137,138], a condition that usually develops in patients previously diagnosed with PD. Dementias are usually considered the result of pathological accumulation of misfolded proteins followed by degenerating pathways of selective synaptic loss [139], although reduced vascular perfusion is emerging as an additional relevant triggering factor [140]. 

Regarding FTD, genetic studies have found mutations associated with the development of the disease in several autophagy-related genes, such as p62 [132], charged multivesicular body protein 2b (CHMP2B), a protein that is involved in the later steps of autophagy and regulates endosomal sorting [141], and Valosin-containing protein (VCP) [141,142]. VCP governs critical steps in ubiquitin-dependent protein quality control in the context of membrane dynamics, with a loss of function mutations found in FTD, resulting in impaired lysosomal clearance and, thus, reduced mitophagy. Of note, the FTD genetic profile has strikingly strong similarities with that of amyotrophic lateral sclerosis (ALS), suggesting that they could be mechanistically connected diseases [143]. In particular, for VCP, and two other mitophagy-related genes, TBK1 and OPTN, mutations have been associated with both FTD and ALS. Consistently, impaired mitophagy has also been demonstrated in ALS patients at different levels.

#### 2.1.3. Alzheimer’s Disease

AD, the most common neurodegenerative disease, is characterized by the accumulation of amyloid-β (Aβ) peptides and the aggregation of hyperphosphorylated Tau protein (pTau), resulting in dysfunctional synapses and neuroinflammation, followed by neuronal loss and clinical symptoms [144]. Although metabolic and mitochondrial alterations are common in all cases, the roles of mitochondrial alterations and mitophagy have been more extensively studied in the context of AD. However, as in the case of PD, since amyloid β (Aβ) plaques have been reported to be the cause of AD pathogenesis, Aβ plaques are considered the main cause of impaired brain metabolism, which may not be always the case. Common findings that relate the altered mitophagy pathway to Alzheimer’s patients include increased granulovacuolar degeneration and pTau deposits, the accumulation of autophagy intermediates, and high levels of aberrant or dysfunctional mitochondria inside the lysosomes [145].

Mitochondrial respiratory chain is generally compromised in AD patients, due to a general deficit in OXPHOS-related enzymes, which impairs metabolic activity in AD brains [146]. Aβ has been shown to directly reduce ETC enzyme activities and disturb mitochondrial respiration. It also influences pathways related to oxidative stress [147]. For example, it binds to a β-binding alcohol dehydrogenase (ABAD) forming an Aβ-ABAD complex, which when inhibited reduces oxidative stress and apoptosis through an undefined mechanism. It has also been shown that Aβ binds cyclophilin D (cypD), a component of the mitochondrial transition pore. The complex apparently also plays a role in the increase in both oxidative stress and apoptosis, since cypD knockout mice with Aβ mutations were able to preserve cognitive function and had decreased oxidative stress and apoptosis. Aβ also disrupts Ca^2+^ homeostasis by stimulating Ca^2+^ transport into the cytoplasm and inhibiting processes to reduce cytosolic Ca^2+^. The high concentration Ca^2+^ in the cytosol affects ATP^+^ production, decreasing OXPHOS. This leads to a depolarization of the mitochondrial membrane, followed by impairment of Ca^2+^ buffering. The Aβ precursor protein (AβPP) has also been shown to block TOMM40 (translocase of the outer mitochondrial membrane 40) activity, which in turn reduces Cytochrome ***c*** oxidase (COX) activity, impairing ATP production [144,148]. Both Aβ and AβPP disrupt the fusion/fission balance in mitochondrial dynamics by increasing fission, leading to neuronal dysfunction [149].

However, accumulated evidence suggests that mitochondrial alterations are also a primary trigger in Alzheimer’s and can impact the production and accumulation of Aβ [150]. Alzheimer’s has been strongly connected to an insufficient mitophagy, since an abnormal increase in autophagic vacuoles (AV) containing deficient mitochondria related to altered PINK1 and Parkin activities has been found. It has been reported that Aβ can bind and deplete cytosolic Parkin, which in turn leads to PINK1 accumulation [151]. As a result, deficient mitochondria are not ubiquitinated. Aβ has also been proposed to induce lysosomal dysfunction [84]. Defective lysosomal proteolysis can lead to increased mitophagosome accumulation and reduce the mitophagy flux [152]. Supporting the relevant role of lysosomes in AD, mutations in two lysosomal genes, Presenilin 1 and apolipoprotein e4 (ApoE4), have been associated with AD development and have been shown to cause lysosomal dysfunction [153]. Presenilin 1 is responsible for lysosomal acidification and ApoE4, a variant of ApoE, destabilizes lysosomal membranes. PINK1/Parkin activity is also affected by the accumulation of AD mutant Tau, which leads to an increase in Parkin sequestration in the cytosol and a reduction in the targeting for the degradation of damaged mitochondria, leading to their accumulation and an overall decrease in basal mitophagy levels [151]. Additional mitophagy-related alterations found in AD patients include reduced Aβ-mediated Disrupted In Schizophrenia 1 (DISC1) activity and DISC1 mutations that result in the defective retrograde transport of mitochondria [86,88,89,90].

In sum, the picture that emerges for PD, AD, and related neurodegenerative processes is that of a context in which mitochondria are largely dysfunctional at least in part because effective activation of mitophagy in response to mitochondrial damage does not take place or is insufficient to compensate for it, leading to the accumulation of largely dysfunctional mitochondria.

#### 2.1.4. Post-Stroke Cognitive Impairment (PSCI)

Another highly prevalent subtype of neurodegeneration is post-stroke cognitive impairment (PSCI). It has been suggested that it is essentially identical to Alzheimer’s disease. Nevertheless, the relative contribution of protein aggregates in this case is still a matter of controversy [154], while the relevance of vascular pathology in the process is now well established [155]. Therefore, PSCI is sometimes considered a type of vascular cognitive impairment (VCI) [156].

Mitophagy has been reported to be induced in brain ischemia, with increases in PINK1 accumulation in the outer membrane of mitochondria and increased Parkin/p62 mitochondrial translocation [157]. During the acute ischemic injury, glucose deprivation, results in a drop in ATP levels, that can directly regulate PINK1-Parkin-dependent mitophagy [158]. Moreover, BNIP3 and NIX activate mitophagy in response to hypoxia [62]. Furthermore, during ischemia, mitochondrial fusion proteins Opa1 and Mfn2 are downregulated, while fission proteins, such as DRP1 and Fis1, are upregulated [159], a situation that also favors mitophagy.

A number of studies also support a relevant neuroprotective role of mitophagy activation in stroke. Of particular interest, it has been shown that melatonin post-stroke neuroprotective activity is partially dependent on mitophagy activation [160] and that rapamycin, an Mechanistic Target Of Rapamycin Kinase (mTOR) inhibitor, activates mitophagy and alleviates vascular dementia [161]. However, other studies suggest that inhibition of mitophagy can also be of therapeutic benefit; for example, it has been proposed that Peroxynitrite (ONOO^-^)-dependent activation of PINK1/Parkin dependent mitophagy that occurs through DRP1 recruitment contributes to cerebral injury following stroke [162]. The picture that emerges is that fine tuning of mitophagy activation seem to be a key event in the chain of events that relieve brain ischemia, since both insufficient removal of damaged mitochondria or excessive degradation of essential mitochondria will cause cell death and the therapy approach should take into account the base line mitophagy status of the patient [163,164,165,166].

### 2.2. Mitophagy in Neurodegenerative Diseases: ALS and Huntington’s Disease

ALS and Huntington’s disease (HD) follow surprisingly similar pathophysiological mechanisms, including neuronal and non-neuronal factors, despite their apparently disparate genetic basis. ALS is a neurodegenerative disease affecting mainly motor neurons, which results in a progressive loss of voluntary muscle function until respiratory arrest due to paralysis. Having a broad spectrum of yet to be classified subtypes, ALS can be considered a syndrome rather than a single disease [167]. HD is a neurodegenerative genetic autosomal-dominant disease affecting medium spiny neurons (MSN) through a mutation in the HTT gene. This mutation leads to progressive motor dysfunctions, such as abnormal voluntary and involuntary movements, and psychiatric, as well as cognitive, impairments [168]. Shared features of ALS and HD include inflammation, mitochondrial damage, oxidative stress, and possibly other metabolic alterations causing weight loss. On a molecular basis, both diseases show reduced PGC-1α activity. PGC-1α is a transcriptional coactivator and master regulator of mitochondrial biogenesis and activity, whose reduced activity leads to impaired mitochondrial hormesis. It acts as a disease modifier in ALS and HD, affecting both canonical and central nervous system-specific pathways. 

#### 2.2.1. Amyotrophic Lateral Sclerosis

Multiple genes have been associated with the development of ALS. The first gene to be identified was *sod1*, encoding Cu/Zn superoxide dismutase (SOD1). Normally working as an antioxidant for O_2_^-^ in the cytosol, the mutant protein is translocated to the mitochondria, leading to the impairment of mitochondrial OXPHOS and oxidative stress, possibly through its interaction with VDAC1, impairing protein and ion exchange [169]. It has also been suggested that mutant SOD1 lowers mitochondrial membrane potential and induces the transport of mitochondria into the soma for degradation or recycling [170]. Detailed analysis of the cellular components involved showed that a key event was the damage and reduced motility of the mitochondria located at the axon terminal [171,172]. Mitochondria travel through the axons via connections with microtubules though motors, such as Kinesin-1, responsible mostly for anterograde transport from the nucleus to the axon terminal (from the − to the + end of the microtubules), and Dynein, responsible for retrograde transport from + to −. Mutant SOD1 has been proposed to alter mitochondrial axonal transport by targeting the anterograde transport machinery or its regulators, as well as the retrograde transport of mitochondria [172]. Retrograde transport alterations can be identified early during pathogenesis and results in the accumulation of mitochondria in axon terminals of motor neurons, while anterograde transport deficits show up at later disease stages [171].

Another type of ALS associated genes code for RNA binding proteins that are also involved in RNA processing, including FUS/TLS (translocated in liposarcoma) and TDP-43 (TAR DNA-binding protein 43) [172]. TDP-43, similar to SOD1, has also been associated with mitochondrial homeostasis and both retrograde and anterograde axonal transport and has also been shown to play a role in mitophagy. Mutations in both TDP-43 and FUS result in reduced Parkin levels and increased mitochondrial damage [172,173]. FUS is heavily involved in genetic material processing, such as the regulation of transcription, RNA splicing and transport, DNA repair, and damage response. Mutant FUS accumulates in the cytoplasm; as a result, the expression of mitochondrial genes is reduced. Additionally, similar to SOD1, mutant FUS accumulates on mitochondria [169]. As noted above, ALS and FTD share strong similarities in the genetic profile, with genetic mutations and deficits. In fact, functional similarities have also been noted, and ALS is no longer considered a disease solely restricted to motor neurons, since it also affects cognitive functions. As a matter of fact, one subtype of ALS that has been shown to display characteristic FTD symptoms is caused by mutations in FUS and TDP-43, two genes previously linked to FTD [174]. Furthermore, as noted above, the mitophagy-related factors VCP, TBK1, and OPTN have been found to be mutated in both FTD and ALS [90,175], similar to mutant OPTN. OPTN mutations are rare but have a great functional impact on autophagy and mitophagy. TBK1, which works along with OPTN to enhance mitophagy and ubiquitin binding, when mutated in ALS patients, results in the blockade of autophagosome formation. Inhibition of the mutant forms TBK1 and OPTN, and downregulating PINK1 and Parkin improves mitochondrial homeostasis in these patients [60,176,177], suggesting that in this context the upregulation of PINK1 and Parkin, by increasing the levels of mutant TBK1 or OPTN, enhance the pathological disruption of the mitophagy flux [178]. 

Mutations in other genes associated with vesicular trafficking have also been found associated with ALS. One of those is Alsin, a guanine-nucleotide exchange factor (GEF) that regulates endosome-autophagosome transport and whose WT form has been proposed to protect neurons from SOD1 toxicity, while mutations in Alsin cause an early-onset form of ALS [179]. Another gene associated with ALS is C9orf72, another GEF protein, which regulates autophagy by indirectly inducing the phosphorylation of phagophores in order to form autophagosomes [180]. It is also involved in endosomal trafficking and regulates actin dynamics in motor neurons. RNA transcribed from a mutant form of this gene accumulates and binds RNA-binding proteins, which in turn impairs RNA processing. The resulting mutated RNA, when translated, forms toxic dipeptide-repeat polypeptides (DPRs), which are possibly involved in the induced mitochondrial damage. Mitochondria, in this context, show reduced membrane potential and an elevated production of ROS [169].

Additionally, p62 is mutated in ALS [90]. Mutated p62 is found associated with a variety of pathological protein aggregations, but most importantly it fails in its capacity to work as an adapter for LC3 ubiquitin binding. Pathological mutations localize in the LIR sequence of the protein; as a result, targets with LC3 are not recognized correctly and, as such, are not sufficiently incorporated into an autophagic vesicle (AV), leading to their incomplete degradation. Therefore, the current model for ALS disease progression presumes that both the accumulation of AVs, which can facilitate the aggregation of misfolded proteins, and that of damaged mitochondria compromise cellular well-being [181].

#### 2.2.2. Huntington’s Disease

HD is mainly associated with the effects of mutant HTT (mHtt) bearing an expansion of its polyQ domain that results in protein aggregation affecting proteostasis, axonal transport, transcription, and translation, as well as mitochondrial and synaptic function [182]. On a macroscopic level, it affects striatal medium spiny γ-aminobutyric acid (GABA) neurons (MSN) sequentially—first by loss of the indirect pathway MSNs, which induces hyperkinesis, and second by loss of the direct pathway MSNs, which triggers hypokinesis. The molecular basis for this selectivity has yet to be fully comprehended. Nevertheless, the most accepted current model attributes a relevant role to dopamine D2 receptors in the process, since they are only expressed in the indirect pathway MSN [167]. Similar to other neurodegenerative diseases, HD is accompanied by reduced levels of PGC-1α and mitochondrial biogenesis [181,182], as well as increased oxidative stress and mitochondrial damage. An evaluation of the activity of the master regulator of mitochondrial biogenesis and activity, PGC-1α in the presence of mHtt led to the unanticipated discovery that mHtt directly binds PGC-1α, leading to its inactivation, which largely accounts for the mitochondrial dysfunction observed in this context [183]. Further analysis led to the identification of other proteins whose activity was also directly modified by mHtt. In particular, it has been suggested that mHtt polyQ tracts interact with glyceraldehyde 3-phosphate dehydrogenase (GAPDH), a dehydrogenase generally involved in glycolysis, resulting in a reduction in GAPDH-induced micromitophagy, a process that directly engulfs damaged mitochondria without the need of autophagosomal formation [182,184], while it has also been presumed that glycolysis might also be impaired in HD. It has also been proposed that this interaction results in the cytotoxic translocation of GAPDH to the nucleus [185]. 

HD is also characterized by the accumulation of damaged mitochondria, along with decreased mitophagy flux. This, at least in part, could be related to the observed interaction of mHtt with the autophagosomal target recognition system. HTT functions as an enhancer for selective autophagy target recognition by assisting p62 binding to ubiquitin, but the polyQ expansion in mHtt results in its altered interaction with p62, resulting in the inhibition of target recognition and hence mitophagy [186,187]. mHtt also adheres to other cellular membranes and polyubiquitinated aggregates, thus generally inhibiting recognition by autophagy receptors [188]. Another pathway that could be related to the accumulation of damaged mitochondria in HD is the autophagosomal axonal transport through HAP1, a protein that together with HTT regulates autophagosome dynamics and transport. mHtt disrupts the formation of the complex, affecting both retrograde and anterograde motors, simultaneously or individually, resulting in the malfunctioning of autophagic degradation [189]. Paradoxically, not only mitophagy impairment but also its partial over-activation can also contribute to disease development. VCP binding to mHtt leads to its accumulation and to the induction of mitophagy in a PINK1/Parkin-independent manner, resulting in mitochondria depletion. This process can be prevented through HV-3, the dependent blockade of VCP translocation to the mitochondria [190]. In sum, autophagy in HD progression shows several different pathological alterations, including the hyperactivation of mitophagy and the impairment of autophagic target recognition and transport. All of these result in an accumulation of dysfunctional components, which worsen the clinical phenotype over time. 

All in all, both ALS and HD are strongly related to alterations in mitophagy. The malfunctioning transport of autophagosomes and target recognition seem especially involved in disease progression and could possibly serve as potential targets for pharmacological interventions.

### 2.3. Mitophagy in Developmental Neurodegenerative Diseases (Autism and Epilepsy)

#### 2.3.1. Autism

Autism is often used as a general term for any type of condition related to autism spectrum disorders. The condition in general is a developmental impairment of brain function. It has different levels of severity, Asperger’s syndrome being one of the milder forms of the disorder and autistic disorder, which is very commonly used as a synonym for all conditions on the spectrum, being on the severe side. General clinical symptoms of autism include impairment of interpersonal connections, language, communication, imagination, and a loss of intellectual and behavioral flexibility, represented by repetitive and stereotypical behavior, forming during the first months or years of life [191,192].

Mitochondrial malfunction is a common observation in autism [193]. The activity of mitochondria is generally low in terms of the ATP^+^-coupled oxygen consumption rate, and at the cellular level the mitochondria appear extensively fragmented, accumulating around the nucleus, which leaves synapses without sufficient mitochondria to function correctly [194]. In line with these observations, the levels of the key modulators of mitochondrial fusion, MFN1/2 and OPA1, are generally reduced, while the levels of the fission regulators Fis1 and Drp1 are increased, which results in an increase in fissed mitochondria, localized in the soma of neurons [195]. Likely because fission promotes mitophagy, the accumulation of fissed mitochondria has also been found to be associated with reduced mitophagy in this context. In fact, different components in the mitophagy-related pathways have been shown to be pathologically decreased, resulting in the retention of damaged mitochondria. The PINK1/Parkin-dependent mitophagy process is generally found to be impaired, commonly due to a strong augmentation in PINK1 expression and significantly lower Parkin transcription. Furthermore, WD Repeat And FYVE Domain Containing 3 (Alfy/WDFY3), a protein associated with selective autophagy, has been identified as an autism risk gene [196]. Changes in Alfy/WDFY3 activity are reportedly affecting LC3 lipidation and, consequently, by altering the progression of autophagy-associated complexes, autophagosome biogenesis and target selection. The activation of micro-mitophagy in this context is insufficient to compensate for this loss of macro-mitophagy, resulting in the accumulation of damaged mitochondria, a phenomenon that could also be related to Alfy/WDFY3′s role in micro-mitophagy [196]. All in all, it can be said that mitochondrial malfunction in autism is likely to play a relevant role in its pathogenesis, especially through the impairment of the correct energy production necessary for physiological processes through either the impairment of mitophagy or the excessive fission and accumulation of malfunctioning mitochondria.

#### 2.3.2. Epilepsy

Epilepsy is a complex, multifactorial disease, generally defined by its main symptom, which is frequent and repetitive seizures happening at random, and is subdivided into two syndrome categories: generalized and partial or localization-related. It is strongly associated with an increased incidence of comorbid conditions, such as anxiety, depression, cognitive impairment, and sudden unexpected death [197]. Generalized epilepsy has a strong genetic base and is associated with seizures coinciding in both brain hemispheres, with a mostly normal neurologic function, triggered by a variety of different internal or external stimuli, whereas partial or localization-related epilepsy is represented by seizures in one or more specific locations in the brain that are then able to spread throughout the whole brain. It is caused by one or more triggers of the central nervous system of unknown origin, or due to pathologies in the brain, including some related to metabolic disorders [181]. Epilepsy is common in subjects with autism, although it is highly variable and depends on different factors, such as age, cognitive level, and type of language disorder [198]. It has also been reported that subtle maldevelopment of the brain might affect the occurrence of epilepsy in autism, especially in the area of the hippocampus, since it is most commonly involved in epilepsy.

Epilepsy is linked to mitochondrial alterations mainly because it is a common manifestation of mitochondrial diseases, with a high prevalence in patients with genetic mutations in mitochondrial DNA (mtDNA) [199]. The best accepted model suggests that, because neurons require high amounts of energy, they are especially vulnerable to mitochondrial ETC deficits, resulting in epilepsy. Mitochondrial ETC deficits are relatively common inborn errors of energy metabolism, with a combined prevalence of 1/5000. They are genetically very heterogeneous. Pathogenic mutations have been reported in all 37 mitochondrially encoded genes and more than 80 nuclear genes. Some examples include mtDNA mutations associated with the mitochondrial encephalomyopathy, lactic acidosis, stroke-like episodes (MELAS), and myoclonic epilepsy with ragged red fibers (MERRF) syndromes). Mutations in *POLG* coding for a mitochondrial DNA-polymerase are classically associated with Alpers syndrome but are also present in mitochondrial recessive ataxia syndrome (MIRAS), spinocerebellar ataxia with epilepsy (SCAE), and myoclonus, epilepsy, myopathy, sensory ataxia (MEMSA) syndrome. Other examples include deficiencies in mtDNA maintenance, deficiencies in Complex I of the respiratory chain, disorders related to alterations in Co-enzyme Q_10_ (CoQ) biosynthesis, and mitochondrial translation, such as *RARS2* mutations [200]. Importantly, seizure-driven secondary mitochondrial damage has also been described in epileptic disorders, including disorders that are mainly of non-mitochondrial origin [201]. Most pathogenic mtDNA mutations associated with epilepsy are located in mitochondrial tRNA genes. These mutations can affect mitochondrial translation to different extents, depending on the amino acid composition of proteins. The most severely affected subunits are Mitochondrially Encoded NADH:Ubiquinone Oxidoreductase Core Subunit 5 (MT-ND5) and Mitochondrially Encoded NADH:Ubiquinone Oxidoreductase Core Subunit 2 (MT-ND2), thus leading to predominant Complex I deficiency.

Importantly, mitochondrial damage is linked to all forms of epilepsy, both genetic and idiopathic. The link between the clinical phenotype and mitochondrial damage is strongly represented in hippocampal sclerosis through heavily impaired Complex I activity. Another sign for mitochondrial alterations, reported in the histology of the hippocampus, is the lack of COX in neurons, the decrease in mtDNA, and the accumulation of somatic mtDNA deletions.

It has been hypothesized that seizures are the possible result of failures in many neuronal processes related to OXPHOS, such as Ca^2+^ homeostasis, the oxidation of transport proteins, such as ion channels and neurotransmitter transporters, excessive excitability, a decrease in plasma membrane potential, and inhibitory interneuron dysfunction [202]. Epilepsy is characterized by perturbations in the GABA-glutamate-glutamine cycle, which regulates how chemical transmitters are released from neurons and then taken up by the supporting cells, the astrocytes. Epilepsy-related alterations include increased extracellular levels of glutamate, loss of astroglial glutamine synthetase activity, and changes in glutaminase and glutamate dehydrogenase. A model has been proposed that links mitochondrial damage with deficiencies in glutamine synthetase and seizure generation processes [203].

Since reduced mitochondrial activity seems fundamental in epilepsy, a role for mitophagy has also been proposed. Evidence of the association of mitophagy with epilepsy includes a study that used kainic acid (KA)-induced status epilepticus (SE) in rats [17]. KA is a highly excitatory glutamine acid analog. This model showed increased mitophagy triggered by the accumulation of succinate and oxidative stress, though how or whether this increment in mitophagy contributes to SE has not described. Cellular models of the myoclonic epilepsy with ragged red fibers (MERRF) syndrome also show AMPK-mediated activation of mitophagy mediators and mitophagy initiation [204]. Importantly, though mitophagy might be triggered correctly, there seems to be a defect downstream leading to an improper autophagy flux, which results in the accumulation of autophagosomes. It has also been proposed that impaired mitophagy might be related to reduced ATP^+^ levels that inhibit lysosomal function and protein sequestration. Furthermore, since various cell models of epilepsy show a deficiency in coenzyme Q 10 (CoQ), supplementation of CoQ has been used as a treatment to alleviate pathophysiological disruptions. Importantly, CoQ supplementation has been shown to result in the enhancement of mitophagy flux via activation of AMPK, which in turn activates Silent Mating Type Information Regulation 2, S. Cerevisiae, Homolog 1 (SIRT1) and PGC-1α, leading to the induction of a metabolic stress resistance program that includes the induction of autophagy [205].

Moreover, it has been demonstrated that epilepsy or epileptic seizures can also result directly from impaired mitophagy. A loss of function of HECT domain and ankyrin repeat-containing E3 ubiquitin protein ligase 1 (HACE1), an that is responsible for ubiquitinating targets for degradation have been connected to disturbed mitophagy flux and a decreased response to oxidative stress [206], which results in spastic paraplegia and psychomotor retardation with or without seizures, leading to epilepsy [207,208,209]. Ceroid-Lipofuscinosis, Neuronal 5 (CLN5) mutations, causing late-infantile neuronal ceroid lipofuscinosis, as well as causing seizures, affect FUNDC1 and p62 activity, two proteins involved in the late steps of autophagy and mitophagy. Furthermore, these proteins have also been found to be dysregulated in a mouse model of the disease [210,211].

In sum, epilepsy does not only show important similarities to other diseases but also informs of the connections among a new set of genes with mitophagy and mitochondrial dysfunction, opening new research venues to target neurodegenerative diseases. Since epilepsy is highly prevalent in autism, it is very likely that children suffering from autism might also be influenced by genetic factors related to epilepsy not commonly associated with autism. These commonalities surely deserve to be further investigated.

### 2.4. Mitophagy in Psychiatric Diseases (Schizophrenia, Bipolar Disorder, and Depression)

Schizophrenia, Bipolar Disorder, and Depression are among the most common psychiatric disorders of the human population, all of them affecting mood and neurologic function. Apart from several important genetic commonalities, these diseases share metabolic alterations as an additional common risk factor. Since mitochondria plays a major role in metabolism, research is currently focused on investigating how alterations in mitochondrial function impact these diseases [212,213].

#### 2.4.1. Schizophrenia

Schizophrenia originates from an interaction of multiple genetic and epigenetic factors that likely disrupt neuronal development during the early stages of life and later in life result in a manifestation of behavioral and cognitive symptoms, likely due to dysfunctional dopaminergic neurotransmission and abnormalities in neuronal connectivity [213,214]. The best characterized susceptibility gene encodes DISC1. DISC1 is a scaffolding protein found abundantly at the spines [215] that has been found to be involved in the regulation of neurodevelopment and neuro-signaling, as well as cell migration, neurite organization, mitochondrial function, and glutamate signaling [216]. It interacts with several proteins involved in intracellular signaling, neurite outgrowth (e.g., Phosphodiesterase 4A (PDE4) and Glycogen Synthase Kinase 3 Beta (GSK3beta)), and synaptic function (e.g., kalirin-7 and TRAF2 And NCK Interacting Kinase (TNIK)) and regulates major signaling and proliferation pathways, such as those dependent on AKT and mTOR activities [217]. Additionally, as noted above, DISC1 also plays a role in mitochondria fusion and fission, and DISC1 mutations are linked to altered mitochondrial transport and activity [218,219,220,221,222,223,224], as well as to impaired autophagy, causing improper degradation or recognition of damaged mitochondria. In schizophrenia, DISC1 and PHB2, another mitophagy-related factor, are commonly found upregulated, causing alterations in mitophagy [219,225,226,227]. Furthermore, DISC1 mutations have been found to be associated with other neurological disorders, such as bipolar disorder and depression, stressing the functional and genetic overlap among those diseases [220]. In schizophrenia, mitochondrial malfunction manifests itself primarily in a poor activity of Complex I, which leads to impaired cellular respiration and mitochondrial dynamics. This is linked to altered energy metabolism and increased oxidative stress, which triggers inflammation and neuronal cell death, supporting disease progression [213,228].

#### 2.4.2. Bipolar Disorder

Bipolar disorder (BD) manifests itself as a biphasic energy shift, i.e., periodically repeated manic and depressive episodes that impair function and cognition and, as such, reduce quality of life [229]. The genetic factors of this disorder have been found to partly overlap with those in schizophrenia. *CACNA1C*, *TENM4*, and *NCAN*, found mutated in both diseases, are known to be neurotrophic molecules in signaling pathways that modulate dendritic sprouting, as well as synaptic and neural plasticity. However, alterations in other pathways involved in neuronal interconnectivity, including mitochondrial function, are also currently being investigated [230,231]. Of particular interest is the malfunctioning regulation of the circadian rhythm and related mitochondrial homeostasis. Accordingly, mitochondrial malfunction is reported to have a great impact on BD pathophysiology. Genes responsible for mitochondrial activity and elements of the ETC show altered expression levels, and lactate levels are elevated due to an elevated glycolytic flux, but phosphocreatine levels are reduced in line with reduced OXPHOS activity. Changes in morphology and the quantity of mitochondria in post-mortem brains have also been reported [230,232]. BD also shows a downregulation in fusion proteins and an upregulation in Fis1, resulting in an increase in fissed mitochondria [233,234]. However, contrary to the assumption that fission is a prerequisite for mitophagy, it actually seems to be downregulated in BD. This effect can also contribute to the observed decrease in OXPHOS, reduced ATP^+^ production, and the accumulation of aberrant mitochondria [232,235]. The resulting metabolic stress activates AMPK, SIRT1, and SIRT3 and elevates pro-inflammatory cytokines and intracellular Ca^2+^, leading to the activation of apoptosis. Since AMPK is directly involved in mitophagy regulation, these changes also impact mitophagy flux in BD. TSPO, a translocator protein that helps transport substances into the mitochondria [236], also seems to play a role in the pathological impairment of mitophagy in BD. An increment in mitochondrial ROS induces TSPO expression, which leads to a decrease in ATP^+^ output, thus inhibiting ubiquitination through PARKIN and subsequently a lack of recruitment of p62 and decreased mitophagy, terminating in the toxic accumulation of mitochondria [237,238]. Evidence on the association between abnormal mitophagy and human neurological and psychiatric disorders has been summarized in Table 1.

#### 2.4.3. Depression

Depression, compared to bipolar disorder and schizophrenia, is more heterogeneous and is more influenced by environmental components than genetics [239]. Depression in itself, either chronic or recurrent, is characterized by depressed mood, anhedonia, feelings of guilt, low concentration and self-esteem, sleep irregularities, increased or decreased appetite, and pessimism. Associated risk factors include the concurrence of a variety of physical disorders, such as CVD, stroke, AD, epilepsy, diabetes, and cancer. However, by itself, it can also be considered as a metabolic, endocrine, inflammatory, or neurodegenerative disorder, a cardiovascular disease, or a deficiency state. It can also result from a lack of sunlight or a dysregulation of the glutamate cycle [239,240]. Mitochondrial function in depression is generally impaired [241]. Low ATP^+^ output rates, and hence energetic limitations for neuronal circuits or signal transduction, as well as imbalanced fusion and fission, oxidative stress, low-grade chronic inflammation, and a pro-apoptotic state, have been reported [241]. DISC1 mutations, as well as mutations p62, the most common genetic component, have been associated with depression. Mutations in p62 impair mitochondrial function [242] and lead to increased anxiety, cognitive decline, depression [243], and the activation of nuclear factor kappa-light-chain-enhancer of activated B cells (NF-κB), driving inflammation [244]. In this context, the forced overexpression of p62 levels results in better mitochondrial function, an improvement in mitophagy, and the maintenance of mitochondrial homeostasis [9,245].

Schizophrenia, BD, and depression, thus, follow similar molecular pathological features, which likely blur the lines of differentiation between these diseases due to overlaps of clinical symptoms. The major feature in mitophagy malfunction seems to be related to mutated DISC1, since it is involved in the impaired recognition of damaged mitochondria through all neuropsychiatric diseases with connections to mood disorders, suggesting that DISC1 might be a new relevant pharmacological target.

**Table 1:** Aberrant mitophagy in neurodegenerative and neuropsychiatric diseases. Disease acronyms are the same as those in the text above. GBA-glucocerebrosidase.

Disease/Mutation	Mitophagy Defect	References
PD PINK1-deficient patients	Excessive mitophagosomes	[89,90,96,97]
PD PARKIN-deficient patients	Abnormal mitophagosomes; blockade mitochondrial turnover	[89,90,95]
Wild-type or PD A53T-Alpha-synuclein overexpression	Reduced mitophagy	[89]
PD L1444 GBA overexpression	Reduced mitochondrial dynamics	[89]
PD SREBF1 mutation	Reduced Parkin levels	[91,92]
PD FBX07 mutation	Impaired mitophagy	[101,102,103]
PD PARK7 mutation	Impaired mitochondria dynamics	[107]
PD LRKK2/PARK8 mutation	Decreased lysosomes, abnormal mitophagy	[116,117,118,119,120]
PD ATP13A2 mutation	Abnormal mitochondria function	[125,126]
PD VPS35 mutation	Reduced parkin activity	[128,129,130]
AD and Down syndrome dementia	Aberrant mitochondria	[137]
AD patients	Aberrant mitochondria Accumulation of autophagy intermediates	[145,146,147,148,149]
AD patients	Impaired Parkin mitochondrial translocation	[84,152]
AD DSC1 mutation	Reduced mitochondria transport	[90]
FTD p62 mutation	Reduced mitophagy Impaired lysosomes	[132,141,142]
ALS patients	Impaired mitophagy	[60,175]
ALS TBK1/OPTN mutations	Disrupted parkin activity	[177]
ALS p62 mutation	Damaged mitochondria Aberrant autophagic vacuoles	[181]
HD patients	Reduced mitochondriogenesis and mitophagy	[182,183,184,185,186,187,188,189]
HD Huntington mutation	Hyperactive mitophagy	[190]
Autism patients	Loss of mitophagy	[196]
Epilepsy HACE 1 mutations	Impaired mitophagy	[206,207,208]
Epilepsy CLN5 mutations	Impaired mitophagy	[210]
Schizophrenia DISC1 overexpression	Altered mitophagy Blocked mitochondrial transport	[219]
BD mutations	Reduced mitophagy Increased fission	[231,232,233,235]
Depression DISC1/p62 mutations	Reduced mitophagy Impaired mitochondrial function	[9,241,242,243,244]

## 3. Mitophagy in Liver Diseases

### 3.1. Non-Alcoholic Fatty Liver Disease

Non-alcoholic fatty liver disease (NAFLD) is the characteristic liver disease associated with metabolic syndrome [246]. When white adipose tissue reaches its expandability limit, which is reduced in the context of systemic low grade inflammation, circulating lipids accumulate and, through reverse lipoprotein traffic, enter the liver, where they accumulate [247,248]. Hepatic lipid accumulation (steatosis) is considered a benign condition, but the high levels of free fatty acids under over-feeding conditions in which mitochondrial activity is relatively low lead to lipotoxicity, where mitochondrial fatty acid oxidation produces high levels of ROS and an accumulation of toxic lipid metabolites [249]. As a result, in NAFLD we find enlarged/swollen mitochondria with reduced cristae and ETC activity, termed mega-mitochondria [248,250].

In order to avoid the loss of functional liver tissue, it is crucial to eliminate injured mitochondria. However, there is evidence that, in NAFLD, mitophagy is significantly inhibited, since accumulated mitophagy intermediates, reduced AMPK activity, and increased levels of mitophagy inhibitors, such as macrophage stimulating 1 (Mst1) or Acyl-CoA:lysocardiolipin acyltransferase-1 (ALCAT1) [251], have been observed. Mst1 is a cell survival regulator associated with liver regeneration and has been found to be upregulated in high-fat diet-mediated fatty liver disease. Increased Mst1 blocks the AMPK pathway and, thus, diminishes Parkin expression, repressing the mitophagy pathway related to Parkin protein. In turn, the pharmacological activation of AMPK restores mitophagy in NAFLD [252]. This pathway, at least in part, involves the inhibition of the mTOR complex and the direct activation of ULK1 [253], the mammalian orthologue of the yeast protein kinase Atg1, which is required for autophagy. ALCAT1 is a lysocardiolipin acyltransferase that catalyzes the pathological remodeling of cardiolipin and is implicated in the mitophagy process. It was initially found to be upregulated in mouse models of NAFLD, where it was found to foster mitochondrial damage and the inhibition of mitophagy. Importantly, ablation of ALCAT1 has been shown to restore mitophagy in an NAFLD model [254], highlighting the relevance of the pathway. Another mechanism proposed to be involved in mitophagy inhibition in NAFLD is the downregulation of a new Parkin-independent mitophagy pathway mediated by the formation of a p62-Keap1-Rbx1 complex. p62 recruits two subunits of a cullin-RING ubiquitin E3 ligase complex, Keap1 and Rbx1, to mitochondria. The p62-Keap1-Rbx1 complex then ubiquitinates mitochondria and promotes mitophagy [251].

### 3.2. Alcoholic Liver Disease

Mitochondrial damage is also caused by other liver disorders [255]. Alcoholic liver disease (ALD) refers to liver damage caused by alcohol overconsumption. Due to the presence of injured mitochondria, Parkin-induced mitophagy plays a protective role against ALD [256], preventing cell death and tissue injury. Mitophagy is activated in response to alcohol consumption by ROS, mitochondrial depolarization, and hypoxia, mediated by BNIP3 and NIX induction [257]. However, the initial adaptive induction of mitophagy fails over time, leading to the chronic maladaptive changes that cause ALD [258].

## 4. Mitophagy in Type 2 Diabetes and Obesity

### 4.1. Type 2 Diabetes

Type 2 diabetes (T2D) is characterized by hyperglycemia and insulin resistance (IR) [259]. These are always associated with mitochondrial damage, likely because high glucose levels enhance mitochondria ROS production and oxidative stress leading to tissue damage [260]. Importantly, mitochondrial ROS have also been shown to play a key role in IR and T2D [261]. Thus, maintenance of mitochondrial quality by mitophagy is crucial in disease development.

Exposure to high glucose levels promotes mitochondrial fission, which is implicated in the mitophagy process, and decreases mitochondrial fusion through Drp1 recruitment and OPA1/MFN degradation, respectively [262]. As a result, T2D patients have smaller mitochondria than in healthy controls [263]. Importantly, despite mitochondrial fission enhancement, an impairment of mitophagy flux in T2D patients has been noted [264]. While subjects with prediabetes accompanied by mild hyperglycemia have been shown to exhibit an increase in the expression levels of several mitophagy-related genes, such as NIX, PINK1, and Parkin, T2D patients have displayed attenuated expression of mitophagy genes [265]. The current model is that increased mitophagy in subjects with prediabetes may result in the elimination of dysfunctional mitochondria, thereby preventing their accumulation and the further aggravation of mitochondrial oxidative stress, while patients with established T2D and higher ROS levels may induce not only an increase in mitochondrial damage but also suppressed mitophagy, thereby resulting in enhanced accumulation of damaged mitochondria [266]. As a result, adaptive increase in mitophagy in subjects with prediabetes may prevent or delay progression to T2D by limiting mitochondrial oxidative stress and damage, supporting the preservation of the β-cell function.

### 4.2. Obesity

Diabetes and obesity are closely related since obesity increases the risk of developing T2D. Therefore, they also show similarities in the role that mitophagy plays in them. Mitochondrial damage has also been described in obesity [267]. In contrast to lean individuals, mitochondria in obese individuals have lower capacity to generate energy, less clearly defined inner membranes, and reduced fatty acid oxidation [268]. Under obese conditions, the mitochondrial fusion and fission balance is disrupted in skeletal muscles by favorably shifting fission mechanisms [266]. However, this change does not induce a better mitophagy response. The evidence shows that, in obese patients, mitochondrial content in the skeletal muscle increases, and mitochondrial biogenesis decreases, suggesting that an obesity-induced increase in mitochondrial content is likely an accumulation of damaged and fissed mitochondria unable to be cleared by mitophagy [267] es 267. These observations possibly suggest that mitophagy would be negatively regulated by excessive fat accumulation or in obese conditions.

Obesity is often associated with white adipose tissue (WAT) abundance and/or brown adipose tissue (BAT) scarcity [269]. The number of mitochondria present in WAT is low compared to that of BAT [270]. Therefore, mitophagy, at least in part, contributes to a whitening of brown adipocytes, turning them into white adipocytes by removing mitochondria [271]). This shows the importance of mitophagy in the transition to obesity-related WAT.

Altogether, although the evidence is that mitophagy is impaired in metabolic disorders, additional research is needed to elucidate the underlying mechanisms involved.

## 5. The Role of Mitophagy in Cardiovascular Diseases (CVD)

In the last decade or so, a significant number of studies have evidenced that alterations in mitophagy can lead to an increased risk of CVD, in particular, to cardiomyocytes, as well as to macrovasculature and microvasculature diseases, such as atherosclerosis and retinopathies [19,272]. Therefore, these studies, as a whole, provide ample evidence that maintaining a proper mitochondrial homeostasis is essential in the cardiovascular system, especially in the heart. Of note, PINK1 is particularly highly expressed in the heart and skeletal muscles.

### 5.1. Mitophagy in Macrovascular Diseases: Atherosclerosis

Atherosclerosis (AS) is a chronic progressive disease and a leading cause of death worldwide [273]. This disease is characterized by the accumulation of lipid-containing plaques in the vascular wall, endothelial dysfunction, vascular smooth muscle cell (VSMC) proliferation, and local inflammation associated with oxidative stress and high apoptotic rates [274,275]. The major clinical consequences of atherosclerosis, such as stroke, myocardial infarction, and tissue ischemia, are due to thrombotic events associated with the acute rupture of an unstable plaque. The stability of the plaque depends on the thickness of the fibrous cap and the degree of cap inflammation. The vascular endothelium plays a central role in atherosclerosis. It is a dynamically adaptable interface that regulates hemostasis, vascular tone, and regulates VSMC proliferation and vascular wall permeability [276]. In addition, the endothelium exhibits anticoagulant and fibrinolytic properties, avoiding platelet aggregation and immune cell adhesion, preventing thrombus formation. Early in disease development, the endothelium is activated by oxidized LDL (oxLDL) deposition, secretes chemokines, and expresses endothelial adhesion molecules, attracting circulating monocytes that transmigrate into the vascular wall intima and differentiate into macrophages [275,277]. When macrophages digest and accumulate oxLDL by endocytosis, transform into foam cells [278], die, and can then create a necrotic core that reduces blood flow. The fibrous cap around the necrotic core can destabilize and detach from the wall, forming a thrombus that can occlude small blood vessels, resulting in ischemia, stroke, and myocardial infarction. Hence, in general, macrophages are viewed as athero-promoting and detrimental elements for plaque stabilization in atherosclerotic lesions. However, several phenotypically distinct macrophages play different roles. While M1 macrophages are pro-inflammatory cells related to plaque rupture, M2 macrophages are inflammation-resolving cells associated with wound healing and tissue repair [279]. Another important cell type in AS disease are VSMCs, the main structural component of the plaque [276]. VSMCs are located in the arterial tunica media and in response to vascular injury or to other stress stimulus. Their response to vasodilators and vasoconstriction mediators is altered, and they switch to a highly proliferative phenotype. Nonetheless, in advanced plaques, VSMCs have shown athero-protective plaque-stabilizing properties, as their apoptosis or senesce promotes cap thinning, resulting in plaque instability [280].

Oxidative stress plays a pivotal role in the progression of the disease and has been associated with mitochondrial malfunction [281]. ROS reduce nitric oxide (NO) bioavailability, reducing vasodilatation and transforming it into peroxynitrite [282], a reactive nitrogen species known to accelerate the atherosclerotic process [283]. Oxidative stress also induces the expression of adhesion molecules in the endothelium that facilitate the adhesion and activation of inflammatory cells and platelets. It also leads to the apoptosis of endothelial cells, the proliferation and dysfunction of VSMCs, and lipid peroxidation. In advanced atherosclerosis, high ROS levels induce the senescence and apoptosis of VSMCs, as well as the formation of abnormal vasa vasorum. Importantly, ROS damage mtDNA, enhancing mitochondrial damage, which increases mitochondrial ROS production and aggravates the oxidative stress state [284,285]. Thus, oxidative stress plays a key role in AS pathogenesis. Regarding inflammation, several studies have pointed out the relation between the uncontrolled inflammatory process and ROS levels, which could promote spreading from a local to a generalized AS [285]. Even though the activation of NOX enzymes is responsible for the large production of ROS in advanced disease stages, mitochondrial damage is a relevant AS [286,287]. There are several studies that show the importance of mitochondrial health in the development of atherosclerosis [288,289]. Mitochondrial impaired activity has been observed in endothelial cells, VSMCs, and macrophages. In fact, abnormal premature primary atherosclerosis in mitochondrial disorders patients (MIDs) in the absence of classical risk factors, called “mitochondrial vasculopathy”, has been observed [272]. In line with these observations, several mutations/deletions in mtDNA have been linked with classical AS [290]. Mitochondrial damage could be measured as increased heteroplasmia rates, due to the accumulation of defective mtDNA molecules bearing both mutations and large deletions. Furthermore, specific heteroplasmic mtDNA variants have been found to be associated with particular atherosclerotic phenotypes [287,288,289,291,292]. Some of them are missense mutations, located in cytochrome c oxidase (COX), nicotinamide adenine dinucleotide hydride (NADH^+^)-dehydrogenase subunits, and cytochrome *b* genes. However, the demonstration that these mutations have a causative role in disease development is still pending. In another revealing study, the reduction in leukocyte mtDNA, possibly due to increased ROS production, could be associated with the severity of coronary AS. 

Mitochondrial damage could also be associated with endothelial dysfunction, a driving factor of AS. It has been found that the mitochondrial m.3243A>G mutation that results in ETC Complex I deficiency [291] confers pro-atherogenic and pro-inflammatory properties to endothelial cells. These properties, in combination with high levels of ox-LDL, induce monocyte adhesion, migration, and transformation into macrophage foam cells [293,294]. In sum, mitochondrial malfunction seems to play a central role in the development of AS, favoring the progression of the disease, and it has even been considered as the initiating factor.

Several studies evidence that mitophagy, by eliminating damaged mitochondria in plaque macrophages, VSMCs, and endothelial cells, helps to reduce cell damage and to maintain plaque integrity, preventing disease progression caused by plaque tissue rupture [295]. The risk of plaque rupture is increased by cap thinning due to VSMC death, as well as collagen and extracellular matrix degradation. In fact, the PINK1/Parkin pathway has been reported to be upregulated in atherosclerotic patients and disease models when compared to normal tissues [295]. For example, multiple studies have shown increased levels of PINK1 and mitophagy in plaque VSMCs, most likely induced by ox-LDL [296,297]. In particular, in VSMCs, mitophagy seems to play an important role in the modulation of cell proliferation and survival. PINK1/Parkin-mediated mitophagy has been shown to regulate VSMC proliferation activating AMPKα, both in vitro and in atherosclerotic plaques in vivo [298]. AMPKα as an energy stress sensor plays a central role in cell growth and survival [299]. Thus, PINK1 deficiency has been shown to result in defective mitophagy and attenuates VSMC survival, while PINK1 and overexpression have been shown to enhance the cytoprotective effect of mitophagy on VSMCs. All this evidence supports the role of mitophagy as a safeguard mechanism against atherosclerotic stress-induced VSMC apoptosis [300,301].

Macrophages and foam cells are key in the development of the disease. However, as noted above, not all macrophage phenotypes are detrimental for AS progression. Mitophagy, and in particular the Parkin target gene NIX, has been proposed to play an important regulatory role in macrophages, contributing to the polarization of macrophages to the M1 phenotype [302]. M1 macrophages have a predominantly glycolytic metabolism, whereas M2 macrophages do not respond to NIX-dependent regulation, due to their dependence on oxidative phosphorylation. It has been established that NIX-deficient macrophages show a decreased expression of pro-inflammatory cytokines and inflammatory regulatory genes, such as *TNFα*, *IL1β*, and *NOS2*, suggesting a causative role for mitophagy in inflammation and, as a consequence, AS progression. However, another study has shown that NIX can play a protective role in arterial vessels, inhibiting the activation of caspase-1 and the maturation of IL-1β induced by ox-LDL in macrophages, possibly inhibiting pyroptosis (ROS and caspase 1-dependent cell death) by decreasing ROS production, and inhibiting NLR Family Pyrin Domain Containing 3 (NLRP3) inflammasome activation [302], suggesting that NIX-mediated mitophagy may delay the development of AS.

In sum, mitophagy in AS is likely to act as a safeguard mechanism, and impaired or inefficient mitophagy can lead to disease progression, promoting endothelial dysfunction and increasing VSMCs death, cytotoxicity, and macrophage polarization to a pro-inflammatory phenotype.

### 5.2. Mitophagy in Heart-Related Diseases: Heart Failure, Ischemia-Reperfusion Injury, and Cardiac Hypertrophy

In order to support the pumping function of the heart, mitochondria occupy almost 30% of the cardiomyocyte volume and produce 6 kg/day of ATP [303,304]. This high metabolic demand implies a high dependency on mitochondrial function. As noted above, ample evidence demonstrates that mitochondrial structural and functional abnormalities contribute to several types of CVD [305]. Damaged mitochondria produce less ATP^+^ and generate dangerous amounts of ROS. Accumulated ROS may damage mtDNA, membrane lipids, and respiratory complex proteins, leading to a catastrophic feed-forward cycle of oxidative damage and ultimately cell death [306]. It is well established that the heart is particularly sensitive to oxidative stress. In fact, chronic ROS exposure in the heart has been related to enhanced ischemia/reperfusion injury (IRI), heart failure (HF), and an increase in different chronic damaging processes, such as apoptosis and fibrosis. Importantly, mitochondrial ROS have also been reported to drive mitophagy induction in the heart, as an emergency response aiming to remove damaged mitochondria [307]. There is evidence that link alterations in the mitophagy with heart disease, highlighting that maintenance of an adequate mitochondrial clearance rate is crucial for cardiomyocyte viability. In fact, reduced numbers of functional mitochondria and/or an accumulation of damaged organelles have been extensively linked to cardiac dysfunction and cardiomyocyte death [308,309]. Therefore, mitophagy is emerging as a potential therapeutic target, particularly in this context, even though it still remains unclear whether mitophagy activation retards or accelerates cardiac damage in CVD.

#### 5.2.1. Heart Failure

HF occurs when an abnormality of cardiac structure or function leads to a chronic, progressive condition where the weakened heart is unable to maintain blood flow to supply the cells with enough nutrients and oxygen, which might result in fatigue and shortness of breath. At first, the heart, pumping faster and stronger, tries to make up for the body’s needs by enlarging and increasing muscle mass (hypertrophy) [310]. HF can be classified as acute or chronic. Chronic HF is a long-term condition characterized by gradual loss of heart contractile capacity and is kept stable by the treatment of symptoms and modification to the patient’s lifestyle. Acute HF is the rapid onset or worsening of chronic HF symptoms, which could be due to HF complications, such as arterial obstruction and derived ischemia [311]. Mitochondrial malfunction is a common pathophysiological phenomenon leading to HF, and studies have revealed that insufficient mitophagy aggravates heart injury, while infarction-induced mitophagy is generally considered as a beneficial homeostatic response to protect the heart [312]. Several studies have analyzed the role of the main regulators of mitophagy in the heart, including PINK1 and Parkin, and the results as a whole support the general conclusion that impaired PINK1/Parkin-dependent mitophagy can work as a driver leading to myocardial dysfunction [38,313]. Although the functions of these proteins overlap, they also have additional and distinct activities in the myocardium. As a result, PINK1- and Parkin-deficient mice have very different cardiac phenotypes. PINK1 appears to be important in maintaining mitochondrial function and redox homeostasis under baseline conditions, as Parkin deficiency had no effect on mitochondria or cardiac function in mice under normal conditions [313,314]. PINK1-deficient mice developed cardiac dysfunction and hypertrophy by 2 months of age. Importantly, PINK1 downregulation and inefficient mitophagy have been described in individuals with HF [315,316]. However, it still remains to be established whether PINK1 deficiency is the cause or effect of heart failure in the human disease. In mice, Parkin-deficient cardiomyocytes have disorganized mitochondrial networks and smaller mitochondria, which elevates the risk of heart failure and makes Parkin knock out (KO) mice more sensitive to myocardial infarction (MI). Reduced mitophagy also contributes to HF by failing to activate mitochondrial DNase II, increasing the levels circulating damaged mtDNA that has not been properly degraded, and leading to the activation of Toll-like receptor (TLR) 9-mediated inflammatory responses [317]. This sterile inflammation in cardiomyocytes can induce myocarditis and dilated cardiomyopathy, contributing to the development of HF.

#### 5.2.2. Ischemia-Reperfusion Injury

Ischemia-reperfusion injury (IRI) is an irreversible adverse effect due to coronary circulation restoration after an ischemic episode. IRI induces mitochondrial fragmentation and stimulates apoptosis in cardiomyocytes [318] and has been shown to activate mitophagy [319]. Its role remains controversial. Mitochondrial oxidative stress per se has also been found to play a central role in the developing of IRI. Importantly, it has been demonstrated that SIRT3, by reducing ROS production and preserving mitochondrial function [320], protects the heart against acute IRI [321]. IRI induces mitochondrial fragmentation through the induction of mitochondrial fission. That, in turn, affects mitophagy because of its inter-dependence with mitochondrial dynamics [194].

Drp1 has been shown to play a key role in cardiac function [322]. Cardiomyocyte-specific Drp1 KO mice shows an accumulation of damaged mitochondria, along with decreased mitophagy activity, which results in cardiac dysfunction and enhanced risk of IRI. Furthermore, cardiac-specific Drp1 HET mice also exhibit a significantly greater infarct size after IRI when compared to WT animals [322,323]. Hence, the tight balance between mitochondrial fusion and fission is essential to conduct a proper mitophagy and is key in the development of IRI. More direct evidence also suggests that mitophagy protects myocardial cells during IRI, eliminating damaged mitochondria before they can hydrolyze an excessive amount of ATP, release pro-apoptotic factors, or produce a high amount of ROS that can lead to HF [324,325]. However, other studies have also shown that the inhibition of mitophagy can potentially protect the myocardium against IRI, decreasing the excessive mitophagy rates caused by Ischemia-reperfusion (I/R), which might preserve mitochondrial integrity and reduce cardiomyocyte apoptosis, improving cardiac function [326]. It has been found that activation of G protein-coupled estrogen receptor 1 (GPER) reduces Parkin translocation from the cytosol to mitochondria, reducing protein ubiquitination and mPTP opening and protecting mitochondria from degradation.

Of note, both PINK1 and Parkin are upregulated during IRI in mice, and this upregulation is necessary to induce ischemic preconditioning (IPC) [327]. IPC is an experimental procedure that drives the induction of a protective cellular mechanism through exposure to brief periods of ischemia. In the heart context, it has been shown to elicit a protective effect against the damage induced by prolonged ischemic episodes, reducing the infarct size and the severity of IRI. It has been shown that Parkin KO mice are resistant to preconditioning [328], and PINK1-deficient mice have been demonstrated to have an increased susceptibility to IRI. Hence, regulation of PINK1 and Parkin gene expression has been proposed as a therapeutic strategy to reduce IRI.

Other mitophagy regulators that have been demonstrated to be relevant in the context of heart IRI: FUNDC1 and Pgam5. FUNDC1 has been found to regulate mitochondrial homeostasis, protecting the heart from IRI [329], while IRI induces FUNDC1 downregulation [330] and promotes FUNDC1 deactivation by inducing the expression of casein Kinase 2 (CK2α), decreasing mitochondrial receptor-mediated mitophagy and enhancing tissue damage. Pgam5 is a mitochondrial serine/threonine protein phosphatase involved in mitophagy at different levels. Pgam5 KO mice have an increased infarct size, which has been correlated with mitophagy inhibition [331]. 

In summary, although the exact role of mitophagy in IRI is still unknown, the accumulated evidence suggests that mitophagy generally acts as a safeguard mechanism and could be involved in maladaptive responses.

#### 5.2.3. Cardiac Hypertrophy

Cardiac hypertrophy is an adaptive compensatory cellular mechanism. It is considered “physiological” (non-pathological) when it is associated with normal cardiac function and “pathological” when it is associated with cardiac dysfunction. Accumulating evidence indicates that pathological and physiological hypertrophy differs in the signaling pathways, and pathological hypertrophy has been associated with a reduction in OXPHOS [332]. Pathological hypertrophy is activated in response to stimuli, such as hemodynamic stress, ischemia, or myocardial injury, and involves morphological and functional changes in cardiomyocytes, increasing cell size, which leads to a growth in heart mass. Although initially beneficial because it compensates contractibility loss and improves myocardial function, pathological hypertrophy can lead to cell death and irreversible structural cardiac remodeling, an increased production of pro-inflammatory cytokines, and cell dysfunction [333], which ultimately contribute to the progression of cardiac disease and HF [334,335].

Increased mitophagy is commonly observed in cardiac remodeling processes, but its role in disease development remains controversial. Several studies have described mitophagy as a degenerative pathway and a maladaptive response. For example, some studies have reported that mitophagy activation contributes to negative myocardium remodeling and fibrosis [336,337]. In addition, NIX upregulation has been reportedly described during age-related pathological cardiac hypertrophy [338]. In contrast, other studies have shown that the absence of key components of the mitophagy molecular machinery can also contribute to pathological cardiac remodeling. In particular, Parkin KO mice present increased cardiac remodeling when compared to WT mice, and PINK1 KO mice show abnormal cardiac mitochondrial function and elevated oxidative stress with early left ventricular dysfunction and pathological cardiac hypertrophy [339]. BNIP3 KO mice have also been shown to develop cardiomegaly at 60 weeks of age. In sum, both down- and upregulation of mitophagy seems to have detrimental effects in the development of hypertrophy and cardiac remodeling. Thus, more research is needed to completely understand the role of mitophagy in this pathology.

### 5.3. Mitophagy in Microvascular Diseases: The Case of Diabetic Retinopathy

Diabetic retinopathy (DR) is a microvascular complication in diabetes and the leading cause of acquired blindness in working-age adults [340]. It could be considered a neurovascular disease, as it affects both blood vessels and neuroglia [341,342]. Persistent hyperglycemia is considered the primary driving factor, since the efficient control of hyperglycemia significantly alleviates disease development [343], and its benefits persist for some after treatment termination [344]. However, the phenomena named glycemic/metabolic memory evidences that glucose per se is not the only driver of the disease, since it can develop despite optimal glucose control. Several studies have reported metabolic modifications in diabetes that are not reversed after glucose normalization [345,346]. Importantly, accumulated evidence shows that mitochondrial damage and mitochondrial oxidative stress persists in T2D patients under optimal glycemic control and is sufficient to drive retinopathy development [346,347,348]. This persistence has been attributed to the epigenetic changes induced by hyperglycemia, which induce changes, for example, in the expression of genes responsible for the protection of mtDNA from damage [345,349]. In general, mitochondria copy numbers are decreased in T2D, and mtDNA is damaged, resulting in impaired transcription of mtDNA-encoded genes and in a compromised ETC [347,349,350,351,352]. For example, the activity of ECT Complex III has been found to be impaired in the retina’s capillary cells [353], while mitochondria showed an increased ROS production and the levels of antioxidants (i.e., MnSOD, Glutathione Peroxidase (GPX), catalase) were reduced [354,355], which makes diabetes a high oxidative stress environment. Oxidative stress damages mitochondria, aggravating and propagating a vicious cycle of mitochondrial damage and ROS production. Increased oxidative stress has been demonstrated in retinal capillary cells (both endothelial cells and pericytes) [356,357] and in nonvascular cells (Müller cells and photoreceptors) in response to high glucose levels and antioxidants, which have demonstrated beneficial effects in ameliorating the development of DR, evidencing the central role of oxidative stress in this disease [355,358]. The retinal susceptibility to oxidative damage can be related to its particular fatty acid composition.

In line with these observation, a large number of studies have shown that T2D is associated with the presence of damaged and swollen mitochondria accumulating in the retina, with partial crystolysis and impaired respiration [349,355,359,360,361,362]. In sum, retinal mitochondria are dysfunctional in diabetes [360], but the extent to which this is the cause or the consequence of the disease remains unclear.

Mitochondrial function in DR has also been shown to depend on the regulation of autophagy, and DR progression has been found to be negatively correlated with mitophagy. When damaged mitochondria are not properly removed by mitophagy, they lead to retinal capillary cell (pericytes and endothelial cells) apoptosis and to ROS increase that contribute to a metabolic switch, changing the activity of GAPDH in glycolysis, increasing the formation of toxic AGEs (advanced glycation end products), and leading to the activation of PKC, which is associated with retinal inflammation, cell loss, and microvascular dysfunction [361,363], all of which promote the development of the disease and finally blindness.

As noted above, ROS have been reported to play a pathogenic role in DR, but they also generally activate mitophagy, which is known to plays a protective role in DR. This apparent paradox could be related to the observed inhibition of mitophagy by via an ROS-mediated inactivation of the PINK1/Parkin signaling pathway [364]. This effect is dependent on the activation by high glucose (HG) of the thioredoxin-Interacting Protein (TXNIP), a protein that binds to thioredoxin (Trx) and inhibits its antioxidant activity, triggering cellular oxidative stress [365,366], as observed in diabetic rat retina (in vivo and in vitro) and in retinal endothelial cells in culture [366,367,368]. Enhanced levels of TXNIP have also been demonstrated to induce mitochondrial damage and reduced mitophagy in retinal Muller cells, where TXNIP has been shown to be involved in Parkin-dependent ubiquitination and targeting damaged mitochondria to lysosomes during mitophagy [366,369].

It is important to mention here that mitochondrial function and cycles of fusion–fission are closely interconnected. Both fission-promoting Drp1 and fusion regulators, such as MFN1/2, are energy-dependent GTPases [370,371]; as such, they need a correct mitochondrial function to work properly. However, while the loss of mitochondrial fission has no overt deleterious effect, defects in fusion are associated with multiple metabolic and epigenetic modifications, such as changes in DNA methylation patterns. In particular, MFN2 deficiency has been reported as lethal [371]. Thus, alterations in the fusion–fission machinery [372] are a central part of mitochondrial alterations in diabetes that also directly impact mitophagy. It has been shown that retinal microvasculature had decreased expression levels of MFN2 and increased Drp1 expression [373]. Consistently, diabetic rodents also show increased Drp1 levels in endothelial cells, along with fragmented mitochondria. TXNIP also seems to play a role in this context, as Drp1 association with mitochondria is enhanced by TXNIP. On the other hand, the available data suggests that mitochondrial fusion plays a protective role, since the overexpression of MFN2 in retinal endothelial cells prevents hyperglycemia-induced mitochondrial damage and ameliorates the accumulation of mtDNA mutations [373]. Thus, it has been proposed that MFN2 could be a therapeutic target for DR treatment.

Retinal ganglion cells (RGCs), neurons that project their axons outside the retina, forming the optic nerve, are also key elements in the progression of DR. Of note, several reports indicate that the differentiation of mouse RGCs depends on mitophagy [374,375]. This process is triggered by tissue hypoxia, which increases NIX expression. In fact, the retinas of NIX KO mice show increased mitochondrial mass, reduced glycolytic enzyme expression, and decreased neuronal differentiation [374].

In conclusion, mitophagy and mitochondrial dysfunction could act as therapeutic targets in DR treatment. However, although the role of mitochondria in diabetes is widely known, more research on the role of mitophagy in DR is still required.

## 6. Mitophagy in Skeletal Muscle Diseases

Mitophagy is necessary for myogenic differentiation, as demonstrated both in vitro, using C2C12 myoblasts [376], and in vivo. Mitophagy was demonstrated to be essential for the preservation of satellite cells [377], which are critical precursors necessary for muscle regeneration [378]. Of note, impaired mitophagy in muscle satellite cells has been demonstrated in aged mice and is related to the loss of their quiescent status [379]. Preservation of skeletal muscle performance during aging has been shown to depend on a good quality control of mitochondria [380,381,382,383]. Mitochondria in skeletal muscle fibers present different morphologies, activities, and capacities to respond to changes in energetic requirements, depending on whether their localization is sub-sarcolemmal or inter-myofibrillar. Moreover, mitochondria also differ between red muscles rich in type I fibers with densely interconnected mitochondria and fast twitching white muscles bearing mainly type II fibers with lower mitochondrial content, which are more prone to wasting. Both aging and physical training greatly modify mitochondria turnover and efficiency in this context [383,384]. The capacity to adapt mitochondrial activity to energetic requirements and exercise levels characterizes skeletal muscles in the young and is gradually lost during aging despite increased mitophagy fluxes in the old [385]. However, the preservation of mitophagy in the elderly is induced by exercise and still plays a protective role, as evidenced by a study showing that muscles in physically active aged men had more sustained mitophagy and a proper quality control of mitochondria [386]. Conversely, the accelerated aging mouse model Senescence accelerated mice P8 (SAMP8) showed, prior to overt sarcopenia, reduced mitochondrial quality and dysregulated autophagy and mitophagy [387].

However, the skeletal muscle is normally able to adapt during chronic exercise and modify its mitochondrial pool through the renovation of mitochondria and limited mitophagy [388,389], while chronic muscle inactivity has been associated with abnormal upregulated mitophagy, leading to a permanent oxidative metabolism deficit [390]. Physical inactivity was found to increase mitophagy and reduce mitochondrial biogenesis in a mouse model of hind suspension and in human biopsies of vastus lateralis muscle after 7 days of immobilization [391]. In apparent contrast with these results, chronic contractile activity has been shown to cancel selective mitophagy, inhibit Transcription Factor EB (TFEB) and lysosomes biogenesis, and, thus, preserve resident mitochondria [83,386]. It must be noted that there are muscle-dependent differences in the regulation of mitochondrial balance and mitophagy, as evidenced in a study that compared the responses of the rat gastrocnemius and tibialis anterior muscles to an immobilization and remobilization model [390], where it was found that mitophagy was the most prevalent event in the gastrocnemius (type II) during immobilization and in the tibialis anterior (type I) during remobilization.

Fasting also induces muscle wasting and mitophagy in an Fibroblast Growth Factor 21(FGF21)-dependent manner through the activation of BNIP3 [392]. BNIP3 has also been shown to be a crucial mediator of mitophagy signaling in type 2 muscle fibers in response to lipin 1 [393]. On the other hand, another established condition of disrupted autophagy and mitophagy in skeletal muscle is obesity and the metabolic syndrome often linked to insulin resistance.

Defective mitophagy of abnormal mitochondria is also a hallmark of Duchenne muscle dystrophy and in mdx dystrophin-deficient mice [394]. Similarly, inflammation, mitochondrial disruption, and abnormal mitophagy have been clearly documented in degenerative sporadic inclusion body myositis [395,396].

Several therapeutical approaches targeting mitophagy have already been tested in muscle wasting models. Recent evidence indicates that antioxidant dietary supplementation with resveratrol, a natural polyphenol, restored mitophagy and eliminated dysfunctional mitochondria both in vivo and in C2C12 myotubes [397]. Additionally, urolithin A, a natural food supplement, was tested successfully in vitro and in rodents as a mitophagy activator able to potentiate muscle endurance capacity [398]. In the first human study reported by Andreux et al. [399], urolithin A was tested in sedentary elderly individuals treated for 4 weeks with oral doses of 500 mg and 1 g per day. The results of the study showed improved mitochondrial markers, enhanced fatty acid oxidation rates, and upregulation of mitochondrial genes in the vastus lateralis muscle.

## 7. Mitophagy and Lifestyle Impact on Aging

Mitochondrial functional impairment is a hallmark of aging. During aging, mtDNA mutation and ROS generation increases, and ATP production declines. These effects impact aging and age-related pathologies and may be related, at least in part, to mitophagy. It has been shown that, in *C. elegans*, a widely used model system to study aging, Daf-16/FOXO controlled germline tumor affecting-1 (DCT-1) activity (BNIP3 homolog) induces mitophagy and extends life span [375]. Lifestyle likewise impacts mitochondrial quality control systems, as evidenced by studies showing, for example, how physically active men have a higher expression of mitochondrial fission and mitophagy markers than sedentary men, while overfeeding, a risk factor of such pathologies as CVD and liver disease, is related to the accumulation of damaged mitochondria and oxidative stress [400]. This is likely related to the decreased activity of AMPK, a protein that activates mitochondrial biogenesis and mitophagy in response to low nutrient availability through the activation of ULK1 [401] and PGC-1α, respectively [253]. As indicated before, AMPK also induces SIRT1 activity, an nicotinamide adenine dinucleotide (NAD^+^)-dependent deacetylase, that in turn activates PGC-1α. Of note, the NAD^+^/NADH^+^ ratio has also been shown to be reduced in aging, along with SIRT1 activity, which also negatively impacts mitophagy directly [402].

## 8. Mitophagy in Cancer

Cell proliferation is linked to metabolic reprogramming that suppresses OXPHOS and increases the glycolytic flux. Conversely, cancer cells typically show reduced OXPHOS activity and increased glycolysis [403]. Importantly, mitochondria in cancer cells are not just reprogrammed but are generally extensively damaged, producing high levels of ROS that can contribute to disease development. In this context, mitophagy, as a key process in mitochondrial homeostasis, has been shown to impact both metabolic reprogramming and the accumulation/elimination rates of damaged mitochondria in tumors by different mechanisms, thus, as a whole, playing a key role in cancer cell survival. In the hypoxic tumor environment, Hypoxia Inducible Factor 1 Subunit Alpha (HIF-1α) activates BNIP3 and NIX, resulting in mitochondrial mass reduction due to the increased mitophagy flux, thus facilitating the cancer metabolic switch [404]. However, many mitophagy adaptors have been found mutated or silenced in several cancer types [405], suggesting a tumor-suppressive role for mitophagy.

A loss of PINK1/Parkin or BNIP3 leads to decreased mitophagy fluxes and to the accumulation of damaged mitochondria that generate high levels of ROS that facilitate the stabilization of HIF-1α, while the tumor suppressor and DNA damage sensor p53 induces BNIP3 and promotes mitophagy, thus limiting metabolic rewiring, as demonstrated in head and neck squamous cell carcinoma cell lines following irradiation [406].

## 9. Mitophagy in Inflammation

Metabolic rewiring is also important in immunity. Proinflammatory response by immune cells (M1 macrophages, Th1 and Th17 lymphocytes, dendritic cells, and microglia) is linked to high levels of anaerobic glycolysis, while M2 macrophages and regulatory T cells rely heavily on mitochondrial OXPHOS metabolism [407]. Mitophagy contributes to the regulated metabolic switch towards glycolysis in M1 macrophage polarization in an NIX-mediated manner [408]. Additionally, a loss of PINK1 induces proinflammatory markers in glial cells, suggesting a protective role for mitophagy in inflammation [409]. Additionally, IL-10, an anti-inflammatory cytokine, induces mitophagy, preserves mitochondrial activity, and inhibits the glycolytic flux via mTOR inhibition in macrophages [410]. On the other hand, as noted before, the accumulation of damaged mitochondria triggers inflammasome activation due to elevated ROS production, high cytosol Ca^2+^ levels, and mtDNA release. Thus, NLRP3 inflammasome activity is induced by ROS, and the inhibition of mitophagy enhances ROS production and inflammasome activity [411].

## 10. Targeting Mitophagy

In view of the important role played by mitophagy in highly prevalent chronic human diseases, there is an emerging interest in the pharmacological regulation of mitophagy to treat mitochondria-related pathologies. However, to date, only a few human studies on mitophagy modulators have been reported. The vast majority of available data comes from preclinical studies. Natural compounds, such as pomegranate and *Ginkgo biloba* extracts, or chemical compounds modulators of mitophagy have been tested mainly in vitro [412,413,414]. A new type of molecule initially developed for cancer treatment is attracting significant scientific interest as a promising mitophagy therapeutical tool, the so-called selective, ubiquitin-mediated “autophagy targeting chimera” (AUTAC) [415], which is able to potentiate mitochondrial turnover and facilitate the removal fragmented mitochondria in vitro in fibroblasts [416].

Additionally, some pharmacological drugs and natural compounds with proven and approved metabolic activities have been tested in mitophagy-related studies, and a number of them have been demonstrated to show mitophagy-activating activities that may be related to their mechanism of action and could allow for the repurposing of these drugs for diseases where mitophagy plays a central role, such as Parkinson’s disease. The most important of these are SIRT1 activators, including resveratrol and the hypoglycemic agent and ETC Complex I inhibitor, metformin. However, there are many other well-known drugs and natural compounds known for their mitophagy-activating role and their benefits to health, such as melatonin.

*SIRT1 activators.* Several studies indicate that resveratrol and polydatin, a resveratrol glycoside with a strong antioxidative effect, have cardioprotective effects when administered before a heart ischemic episode [417]. In particular, a study by Ling et al. found that polydatin treatment post-infarctum could reduce myocardial IR injury and myocardial infarct size in mice via mitophagy activation, which allowed for better control of mitochondrial ROS production, cell death, and inflammation [418].

*Melatonin.* It has been described that melatonin can promote mitophagy in an atherogenic mouse model via activation of the Parkin/SIRT3/FOXO3a pathway. The results of this study indicate a significant increase in the LC3II/I ratio and Parkin expression levels and a decrease in TOMM20. As a result, authors have proposed mitophagy induction by melatonin as a therapeutic strategy for the treatment of atherosclerosis [419]. Moreover, another study by Díaz-Casado et al. demonstrated in a zebrafish model of Parkinson, that melatonin treatment could restore the PINK1/Parkin/DJ-1/MUL1 network, activating the mitophagy flux and hence rescuing the zebrafish embryos from Parkinson [420].

*Metformin.* Although metformin is best known for its anti-hyperglycemic effects, its capacity to activate mitophagy has also been proposed to be involved in its therapeutic effects in several disease contexts, including T2D and its complications. A study of Bhansali et al. found that metformin promotes mitophagy via AMPK activation by increasing the expression of key mitophagy genes, such as *PINK1*, *Parkin*, *NIX*, and *LC3*, and preserving mitochondrial health in the mononuclear cells of T2D patients [421,422]. Another study focused on the elucidation of the mechanisms involved showed that metformin could also increase Parkin expression via reducing NF-κB activation and, thus, promoting the mitophagic flux in cultured human renal epithelium exposed to high glucose levels, an effect that could also be indicative of the functional relevance of mitophagy in the context of diabetic nephropthy [423]. In line with these findings, Song et al. reported that metformin treatment facilitates Parkin-mediated mitophagy in an obese mouse model by increasing the degradation of mitofusins and decreasing the inhibitory interaction of cytosolic p53 with Parkin [424]. Of interest, metformin applications related to its capacity to activate mitophagy may be able to stretch far beyond obesity and T2D. Of note, Wang et al. showed that mitophagy activation by metformin via the SIRT3-mediated PINK1/Parkin signaling pathway in primary chondrocytes had protective effects against osteoarthritis [425].

Out of many other compounds whose therapeutic effects have been attributed to the activation of mitophagy-related pathways, some of the most relevant are as follows:

Ursolic and oleanolic acids have been proposed to have an anti-tumorogenic activity that is dependent on their activation of mitophagy. Treated cancer cells showed an increase in ROS production, elevated PINK1 expression, and enhanced recruitment to the OMM, but only oleanolic acid triggered an increase in Parkin, while ursolic acid could also activate mitophagy via the AKT/mTOR pathway [426].

The anthelmintic drug niclosamide and its analogue AM85 (Dibromsalan) activate PINK1 in primary cortical neurons and HeLa cells, triggering the PINK1/parkin mitophagy pathway. Although the mechanism of action of these drugs seems to be indirect via the reversible reduction in the mitochondrial membrane potential, they could have therapeutic potential, slowing down PD progression through the activation of PINK1 [427].

Gerontoxanthone I (GeX1) and macluraxanthone (McX), which have been proposed to elicit protective effects against myocardial IR injury, have been shown to promote PINK1 stabilization and Parkin phosphorylation and induce the degradation of mitophagy-related proteins, such as TOMM20 and TIMM23, inducing mitophagy through the PINK1-Parkin pathway [428].

Notoginsenoside R1 (NGR1), a saponin extracted from *Panax notoginseng*, has been described as a potential candidate for DR treatment based on its capacity to enhance mitophagy and elevate the levels of PINK1, Parkin, and the ratio of LC3-II/LC3-I in the retinas of leptin receptor KO mice (*db/db* mice) [358], a T2D model.

Salidroside (Sal) is a phenylpropanoid glycoside extracted from *Rhodiola* [429]. Several studies support its therapeutic use for the amelioration of intervertebral disc degeneration, suggesting that it works, at least in part, through the activation of Parkin-mediated mitophagy. It has been proposed that Sal promotes Parkin expression, activates mitophagy, scavenges ROS, and suppresses apoptosis [430]. However, Sal may also promote mitophagy in a neural cell line, suggesting that it could be used as a treatment to improve the functional recovery after spinal cord injury.

Other compounds that are showing promising results in inducing mitophagy in different animal models or human cells are urolithin A, a small natural compound extracted from pomegranate [431], the antibiotic actinonin, and NAD^+^ precursors (NMN and NAM) [432]. Several studies indicate that urolithin A and actinonin induce the expression of mitophagy proteins, such as PINK1 and Parkin, in models of AD [84]. The NAD^+^ precursors seem to stimulate mitophagy in a more indirect way, promoting mitochondrial fission and SIRT1-dependent deacetylation of Atg5, Atg7, and Atg8 and the effectiveness of this treatment is being tested in humans.

Nitric oxide (NO) is known to modulate mitochondrial dynamics and biogenesis [433,434]. Consistent with this notion, Han et al. showed that optimum levels of NO were able to induce Parkin translocation to the mitochondria in dopaminergic neuronal cells, even in the absence of PINK1 [435]. Furthermore, in a previous study, Kim et al. found that NO dysregulation was important in PINK1-deficiency-related mitochondrial Complex IV deficits and that NO treatment could help dopaminergic neurons to restore ETC enzyme levels [436]. Therefore, using an NO signaling activator, such as ginsenoside Re or an NO donor, such as sodium nitroprusside (SNP) and S-nitroso-N-acetyl-DL-penicillamine (SNAP), could serve as a novel pharmacotherapy in the treatment of certain forms of PD and other mitophagy-related diseases by activating mitophagy, even in the absence of PINK1. However, more studies are needed to fully elucidate the functional relation between PINK1 levels and NO signaling alterations and how these affect mitochondrial functions.

Finally, in recent years, targeting the de-ubiquitinating enzymes (DUBs) to selective increase mitophagy, even in the absence or loss of function of Parkin or PINK1, has been of increasing interest. One of the most promising targets is the USP family, in particular USP30, a cysteine protease located in the OMM that opposes Parkin-mediated mitophagy [437]. There are several small molecules and natural compounds that could act as inhibitors that are being studied [438]. However, the number of potentially effective compounds is still limited, since some of these, target several DUBs, and others are yet to have been tested in humans.

## 11. Conclusions

The central relevance of mitophagy in all diseases related to metabolic control is now well established. As mitophagy is required to control metabolic homeostasis or remove damaged or unnecessary mitochondria, it prevents mitochondrial malfunction and subsequent molecular events, such as oxidative stress, that lead to disease development. In diseased states, mitophagy can sometimes partially compensate other deficits alleviating them, but when mitochondrial activity is compromised, mitophagy can actually play a detrimental role. This is specially evidenced in diseases where normal mitophagy activity is compromised by genetic or regulatory events. These results have boosted pharmacological research in the field, since there are several potentially druggable targets along the mitophagy pathway. Some of these molecules are already showing promising results, and more will certainly come soon. Several natural dietary compounds, such as polyphenols, flavonoids, spermidine, or trehalose, which restore normal mitophagy fluxes in the elderly [439,440], could help to control the inflammasome and to prevent neurodegeneration having a direct impact on apoptosis and the caspase activation cascade [441,442]. Perhaps more importantly, lifestyle interventions can promote cardiovascular health, boosting mitophagy [443,444]. The future could also bring new findings on novel, non-canonical mechanisms of mitochondrial quality control, such as the recently described mitochondrion-derived vesicles, characterized in hypoxic neurons and cardiomyocytes [445].

## Data Availability

Not applicable.

## Abbreviations

Aβ	amyloid-β
ABAD	aβ-binding alcohol dehydrogenase
AβPP	Aβ precursor protein
AD	Alzheimer’s disease
AGEs	Advanced glycation end products
ALCAT1	Acyl-CoA lysocardiolipin acyltransferase-1
ALD	Alcoholic liver disease
ALS	Amyotrophic lateral sclerosis
AMPK	Adenosine monophosphate activated protein kinase
ApoE4	Apolipoprotein e4
AS	Atherosclerosis
ATG32	Autophagy-related protein 32
ATP^+^	Adenosine triphosphate
AUTAC	Autophagy targeting chimera
AV	autophagic vacuoles
α-syn	𝛼-synuclein
BAT	Brown adipose tissue
BCL2	B-Cell CLL/Lymphoma 2
BCL2L13	Bcl2 like 13
BECN1	Coiled-coil myosin-like BCL2-interacting protein
BNIP3	BCL2/adenovirus E1B 19 kDa interacting protein 3
BNIP3L/NIX	BCL2/adenovirus E1B 19 kDa inter-acting protein 3 like
BP	Bipolar Disorder
BRCA1	Breast Cancer 1
CACNA1C	Calcium voltage-gated channel subunit alpha1 C
CaMKK-β	calcium-dependent protein kinase kinase-β
CHDH	Choline dehydrogenase
CHMP2B	Charged multivesicular body protein 2b
CK2α	casein Kinase 2
CL	Cardiolipin
CLN5	Ceroid-Lipofuscinosis, Neuronal 5
CMA	Chaperone mediated autophagy
CVD	Cardiovascular disease
CoQ	Coenzyme Q10
COX	Cytochrome c oxidase
cypD	Cyclophilin D
DCT-1	Daf-16/FOXO controlled germline tumor affecting-1
DISC1	Disrupted in schizophrenia 1
DJ-1	Parkinsonism Associated Deglycase
DPR	Dipeptide repeat
DR	Diabetic retinopathy
Drp1	Dynamin-related protein 1
ER	Endoplasmic reticulum
ETC	Electron transport chain
FBXO7	F-box only protein 7
FBXW7	F-box and WD40 domain protein only protein 7
FGF21	Fibroblast Growth Factor 21
FIS1	Mitochondrial fission 1 protein
FKBP8	FK506-binding protein 8
FTD	Frontotemporal Dementia
FUNDC1	FUN14 domain containing protein 1
GABA	γ-aminobutyric acid
GSK3β	Glycogen Synthase Kinase 3 Beta
GTPases	Guanosine triphosphate hydrolases
GPER	G protein-coupled estrogen receptor 1
HACE1	HECT Domain And Ankyrin Repeat Containing E3 Ubiquitin Protein Ligase 1
HD	Huntington’s disease
HIF1α	Hypoxia-inducible factor 1 Subunit Alpha
HIV	Human immunodeficiency virus
HF	Heart failure
HG	High glucose
IL-1β	Interleukin 1 beta
IMM	Inner mitochondrial membrane
IPC	Ischemic preconditioning
I/R	Ischemia-reperfusion
IRI	Ischemia/reperfusion injury
KA	Kainic acid
KO	Knock out
LC3	Microtubule-associated protein 1 light chain 3
LIR	LC3-interacting region
L-OPA1	Full-length OPA1
LRRK2	Leucin-rich repeat serine/threonine kinase2
MELAS	Mitochondrial encephalomyopathy, lactic acidosis, and stroke-like episodes
MERRF	Myoclonic epilepsy with ragged red fibers
Mff	Mitochondrial fission factor
MFN	Mitofusin
mHtt	Mutant Htt
MI	Myocardial infarction
MIDs	Mitochondrial Disorders
MiD49	Mitochondrial dynamic protein 49 kDa
MiD51	Mitochondrial dynamic protein 51 kDa
MIM	Mitochondrial inner membrane
MIRAS	Myoclonic mitochondrial recessive ataxia syndrome
MIRO	Mitochondrial Rho
MitoPLD	Mitochondrial Phospholipase D
MSN	Medium spiny neurons
mTOR	Mechanistic Target of Rapamycin Kinase
MEMSA	Myoclonus, epilepsy, myOPAthy, sensory ataxia
MPP	Mitochondrial processing peptidase
MPP+	1methyl-4-phenylpyridinium
mPTP	mitochondrial permeability transition pore
MSNs	Striatal medium spiny GABA neurons
Mst1	Macrophage stimulating 1
mtDNA	Mitochondrial DNA
MT-ND5	Mitochondrially Encoded NADH:Ubiquinone Oxidoreductase Core Subunit 5
MT-ND2	Mitochondrially Encoded NADH:Ubiquinone Oxidoreductase Core Subunit 2
NAD^+^	Nicotinamide adenine dinucleotide
NADH^+^	Nicotinamide adenine dinucleotide hydride
NAFLD	Non-alcoholic fatty liver disease
NCAN	Neurocan
NBR1	Neighbor of BRCA1 gene 1
NDP52	Nuclear domain 10 protein 52
NF-κB	Nuclear factor kappa-light-chain-enhancer of activated B cells
NLRP3	NLR Family Pyrin Domain Containing 3
NO	Nitric oxide
6-OHDA	6-hydroxydopamine
OMM	Outer mitochondrial membrane
ONOO^-^	Peroxynitrite
OPA1	Dynamin-like GTPase optic atrophy 1
OPTN	Optineurin
oxLDL	Oxidized LDL
OXPHOS	Oxidative phosphorylation
PARL	Presenilin-associated rhomboid-like protein
PB1	Phox and Bem1 domain
PD	Parkinson’s disease
PDE4	Phosphodiesterase 4A
PGC-1𝛼	Peroxisome proliferator-activated receptor gamma coactivator 1-alpha
PHB	Prohibitin 2
PINK1	PTEN-induced putative kinase 1
PKC	Protein Kinase C
PSCI	Post-stroke cognitive impairment
pTau	Tau protein
p62	Sequestosome-1
RBX1	Ring-Box 1
ROS	Reactive oxygen species
SAMP8	Senescence accelerated mice P8
SCAE	Spinocerebellar ataxia with epilepsy
SCF complex	Skp-Cullin-F-box ubiquitin E3 ligase complex
SE	Status epilepticus
Serine 65	Ser65
SIRT1	Silent Mating Type Information Regulation 2, S. Cerevisiae, Homolog 1
SLP-2	Stomatin-like protein 2
SMURF1	SMAD Specific E3 Ubiquitin Protein Ligase 1
Sod1	Superoxide dismutase 1
SREBF1	Sterol regulatory element binding transcription factor 1
STEMI	ST-Elevation Myocardial Infarction
TAX1	Trans-activating transcriptional regulatory protein of HTLV-1
TAX1BP1	TAX1 binding protein 1
TBC1D	TBC1 domain family member
TBK1	Tank-binding kinase 1
TDP-43	TAR DNA-binding protein 43
TENM4	Teneurin Transmembrane Protein 4
TFEB	Transcription Factor EB
TIMM	Translocase of the inner mitochondrial membrane
TNIK	TRAF2 And NCK Interacting Kinase
TLR	Toll-like receptor
FUS/TLS	Translocated in Liposarcoma
TOMM	Translocase of the outer mitochondrial membrane
TSPO	Translocator protein
Trx	Thioredoxin
TXNIP	Thioredoxin-Interacting Protein
T2D	Type 2 diabetes
UPS	Ubiquitin proteasome system
USP30	Ubiquitin carboxyl-terminal hydrolase 30
VDAC1	Voltage-dependent anion channel 1
VCP	Valosin-containing protein
VSMCs	Vascular smooth muscle cell
Rab-GAP	Rab GTPase activating protein
RGCs	Retinal ganglion cells
VCI	Vascular cognitive impairment
WAT	White adipose tissue
WDFY3/Alfy	WD Repeat And FYVE Domain Containing 3
WT	Wild type
